# DNA Polymerase IV *dinB* Favors the Adaptive Fitness of *mcr*‐carrying Bacteria Through a Negative Feedback Regulatory Mechanism

**DOI:** 10.1002/advs.202411994

**Published:** 2025-01-31

**Authors:** Haijie Zhang, Xia Xiao, Chenlong Wang, Yurong Zhao, Bo Chen, Xinyuan Ji, Lina Gu, Jie Wang, Zhiqiang Wang, Yuan Liu

**Affiliations:** ^1^ Jiangsu Co‐innovation Center for Prevention and Control of Important Animal Infectious Diseases and Zoonoses College of Veterinary Medicine Yangzhou University Yangzhou 225009 China; ^2^ Joint International Research Laboratory of Agriculture and Agri‐Product Safety, the Ministry of Education of China Yangzhou University Yangzhou 225009 China; ^3^ Institute of Comparative Medicine Yangzhou University Yangzhou 225009 China

**Keywords:** bacteria, colistin resistance, dinB, fitness cost, mcr expression

## Abstract

The plasmid‐borne resistance gene *mcr* drastically undermines the effectiveness of colistin, posing a substantial threat to public health. Although several key plasmid elements that balance *mcr‐1* persistence and bacterial growth are identified, the regulatory interactions between *mcr‐1* and host bacteria remain poorly understood. Using a genome‐wide CRISPRi crRNA library, it is identified that DNA polymerase IV, *dinB*, is essential for controlling the fitness cost associated with *mcr‐1* in *Escherichia coli*. The absence of *dinB* operon enhances *mcr‐1*‐mediated colistin resistance but simultaneously compromises bacterial growth and competitiveness. Meanwhile, *dinB* deficiency mitigates inflammatory response in RAW267.4 cells and enhances bacterial colonization in murine tissues. Further investigation reveals that *mcr‐1* actively upregulates *dinB* expression, with the increased reactive oxygen species induced by *mcr‐1* being crucial for this activation.   These findings suggest that *dinB* modulates *mcr* expression and bacterial fitness via a negative feedback regulatory mechanism. Leveraging this regulatory relationship, a Toxin‐Intein is engineered under the control of *dinB* promoter to selectively target and kill *mcr*‐positive *E. coli* both in vitro and in vivo. Overall, the work uncovers a novel adaptive mechanism underlying *mcr* persistence and provides a precise antimicrobial strategy to combat antibiotic‐resistant pathogens.

## Graphical abstract

This work demonstrates that DNA polymerase IV *dinB* favors the adaptive fitness of *mcr*‐carrying bacteria via a negative feedback regulatory mechanism. Specifically, the elevation of ROS production by intact *mcr* activates *dinB*, which in turn represses the *mcr* expression, maintaining a balance between colistin resistance and bacterial fitness. By harnessing this regulatory relationship, a targeted killing system is engineered to selectively eliminate *mcr‐1*‐positive bacteria.

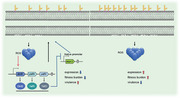

## 1 Introduction

The extensive application of β‐lactam antibiotics has led to substantial bacterial resistance, renewing interest in colistin as a treatment for multidrug‐resistant Gram‐negative infections, particularly those caused by carbapenem‐resistant Enterobacteriaceae (CRE) and Fusobacteriaceae.^[^ ^1^ ^]^ The World Health Organization (WHO) has classified colistin as a “Crucially Important Antibiotic,” designating it as a last‐resort treatment for infections caused by CRE.^[^ ^2^ ^]^ Prior to 2015, colistin resistance was attributed to chromosomal mutations and had not been detected through horizontal gene transfer.^[^ ^3^ ^]^ However, following the discovery of the plasmid‐mediated colistin resistance gene *mcr‐1* in China, various *mcr‐1* gene variants (e.g., *mcr‐1* to *mcr‐10*) have been identified in over ten Enterobacteriaceae species, isolated from diverse sources including animals, food, farms, humans, and the environment.^[^ ^4^ ^]^ Phylogenetic analysis of these *mcr* variants indicated that they likely did not originate from a common ancestor, suggesting the capacity of the *mcr* family for continuous evolution under colistin exposure or other unidentified selective pressures.^[^ ^5^ ^]^ A retrospective investigation traced the presence of *mcr‐1* in databases over the past century, with a notable increase in isolation rates around 2013, posing a serious threat to both human and animal health.^[^ ^4a^ ^]^

Beyond mediating colistin resistance, *mcr‐1* also confers resistance to antimicrobial peptides (AMPs), which are known for their broad‐spectrum antimicrobial activity and play a key role in immune response modulation and pathogen defense. For example, *mcr‐1*‐positive *Escherichia coli* (*E*. *coli*) and *Klebsiella pneumoniae* (*K. pneumoniae*) have shown enhanced resistance to AMPs, including β‐defensins 1–3 and cathelicidin LL37 produced by intestinal epithelial cells, as well as α‐defensins 5 and 6 from Paneth cells.^[^ ^6^ ^]^ Furthermore, *mcr‐1* has been shown to enhance the intestinal colonization and fitness of *E. coli* by downregulating intestinal inflammatory markers and preserving the composition of the intestinal microbiota in both gnotobiotic and conventional mice.^[^ ^7^ ^]^

Notably, the colistin resistance conferred by *mcr‐1* is moderate (typically 2–8 mg L^−1^) in contrast to resistance mediated by chromosomal mutations such as *pmrA*/*pmrB* (typically 32–256 mg L^−1^).^[^ ^8^ ^]^ A previous study indicated that *mcr‐1* overexpression not only impaired growth, cell viability, and competitiveness, but also disrupted cell membrane and cytoplasmic structures, underscoring the necessity for stringent control of *mcr‐1* expression.^[^ ^9^ ^]^ For example, *pixR*, a novel transcriptional regulator gene, enhanced the transcription of 13 essential transfer genes by binding to their promoters, thereby reducing plasmid loss associated with the high adaptive cost of *mcr‐1* and enhancing the invasion and transmissibility of *mcr‐1*‐bearing IncX4 plasmids.^[^ ^10^ ^]^ Additionally, PcnR was crucial for maintaining *mcr‐1*‐bearing IncI2 plasmids in bacterial populations by directly binding to *repA*, balancing *mcr‐1* expression and associated fitness cost by regulating plasmid copy number.^[^ ^11^ ^]^ Furthermore, regulatory evolution in the upstream promoter of *mcr‐1* fine‐tunes its expression to mitigate the fitness burden without compromising colistin resistance, contributing to increased *mcr‐1* stability in pig farms following the prohibition of colistin as a growth promoter.^[^ ^12^ ^]^ Despite these efforts, the interaction and regulatory mechanisms between *mcr‐1* and host bacteria remain poorly understood. Recent studies suggest that chromosomal genes involved in stress response, membrane integrity, and lipid A modification may influence both host fitness and the stability of the resistance trait.^[^ ^13^ ^]^ The potential role of host regulatory networks in modulating *mcr‐1* expression and its subsequent impact on bacterial physiology warrants further investigation.^[^ ^14^ ^]^

In this study, we constructed a genome‐wide CRISPR interference (CRISPRi) crRNA library to identify genetic regulators of *mcr‐1* expression. The results identified DNA polymerase IV, *dinB*, as a pivotal repressor of *mcr‐1* expression. Our subsequent investigations revealed that *dinB* diminishes both transcriptional and translational levels of *mcr‐1*, which in turn enhances bacterial fitness and mitigates the virulence of *mcr‐1*‐positive *E. coli*. Moreover, we elucidated a negative feedback mechanism between *mcr‐1* and *dinB* that is instrumental in maintaining the equilibrium between *mcr‐1*‐mediated resistance and the host's adaptive fitness costs. Collectively, our findings shed a new light on the regulatory mechanisms governing *mcr‐1* expression and may inform the development of targeted strategies to combat antibiotic resistance.

## 2 Results

### 2.1 Construction of Genome‐Wide CRISPRi crRNA Library

**Figure 1:**
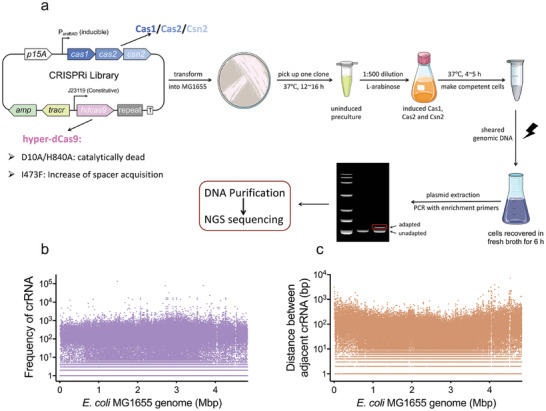
Construction of a genome‐wide CRISPRi crRNA library. a) Construction protocol of CRISPRi crRNA library based on CRISPR‐Cas adaptation system. The CRISPR‐Cas adaptation system comprises p15A replicon, four *cas* genes (*cas1*, *cas2*, *csn2* under the L‐arabinose‐inducible promoter, and *cas9* under the constitutive promoter J23110 modified with three mutations: D10A, H840A, and 473F), a single CRISPR repeat, and the minimal *tracrRNA* (89 bp). “‘R”’ denotes as the location of new spacers integrated into the empty CRISPR array. b) The frequency and location of crRNAs in the library generated by electroporating sheared MG1655 genomic DNA. c) The distance between adjacent crRNAs of a CRISPRi library. The distance was calculated by subtracting the position of the latter crRNA from that of the former one.

We engineered a recombinant *E. coli* MG1655 by integrating the Type II CRISPR‐Cas adaptation machinery from *Streptococcus pyogenes*. This system included four *cas* genes (*cas1*, *cas2*, *csn2* under the L‐arabinose‐inducible promoter, and *cas9* under the constitutive promoter J23110), a single CRISPR repeat, and the minimal *tracrRNA* (89 bp) necessary for crRNA targeting.^[^ ^15^ ^]^ The Cas9 protein was modified with three mutations (D10A, H840A, and 473F) to generate a catalytically inactive variant (hdCas9) with enhanced acquisition rates. Sheared genomic DNA was introduced into the CRISPR‐hdCas system‐overexpressing MG1655 via electroporation, and crRNA adaptation was detected through PCR using enrichment primers (**Figure** **1**a). As shown in Figure S1 (Supporting Information), the PCR product size shifted from 96 bp in unadapted bacteria (Figure S1a, Supporting Information) to 162 bp in crRNA‐adapted cells (Figure S1b, Supporting Information), signifying the successful acquisition of crRNA and an additional repeat. The 162‐bp product from adapted cells was purified and analyzed by deep sequencing, confirming the establishment of a genome‐wide CRISPRi crRNA library. It is noteworthy that high‐fidelity DNA polymerase failed to detect adapted crRNAs during enrichment amplification, necessitating the use of Taq DNA polymerase in the final amplification step. The use of common DNA polymerase increased the likelihood of mutations. While 81.7% of sequenced adapted crRNAs mapped to the genome, only 16.3% of the sequenced crRNA species mapped to the MG1655 genome (Figure S1d,e, Supporting Information). These mapped crRNAs were evenly distributed across the MG1655 genome, with an average gap of less than 100 bp between adjacent crRNAs (Figure 1b,c). These results strongly support the broad applicability of this crRNA‐adapted genome‐wide CRISPRi library.

### 2.2 Identification and Validation of Genes Downregulating *mcr‐1* Expression

**Figure 2:**
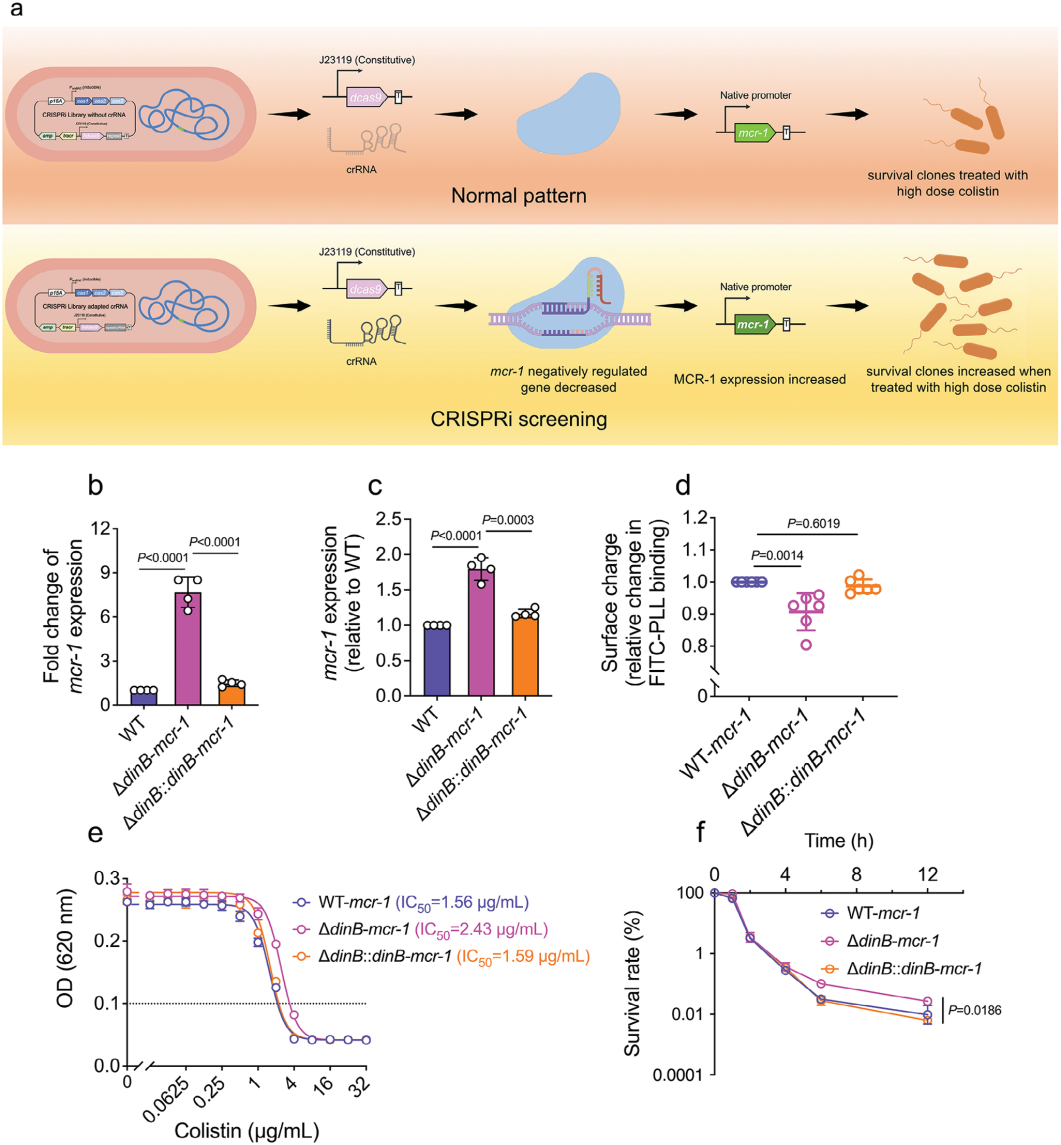
*dinB* reduces *mcr‐1*‐mediated colistin resistance by downregulating its expression. a) Schematic illustration of screening the genes that involved in the regulation of *mcr‐1* expression. b and c) Transcriptional b) and translational levels c) of MG1655‐*mcr‐1*, MG1655‐∆*dinB*‐*mcr‐1*, and MG1655‐∆*dinB*::*dinB*‐*mcr‐1*. d) Relative bacterial surface negative charge measured by FITC‐PLL. Surface charges were calculated relative to MG1655‐*mcr‐1* (set as 1). e) Absorbance of OD_620 nm_ of MG1655‐*mcr‐1*, MG1655‐∆*dinB*‐*mcr‐1*, and MG1655‐∆*dinB*::*dinB*‐*mcr‐1* with increasing concentrations of colistin. f) Survival rates of MG1655‐*mcr‐1*, MG1655‐∆*dinB*‐*mcr‐1* and MG1655‐∆*dinB*::*dinB*‐*mcr‐1* upon 1 µg mL^−1^ colistin treatment. *p*‐values were determined using an unpaired, two‐tailed Student's *t*‐test.

To elucidate the regulatory mechanisms governing *mcr‐1* expression, we utilized our previously established genome‐wide CRISPRi library to identify genes that repress *mcr‐1* function. Silencing specific genes via the CRISPRi library reduced their repressive effects on *mcr‐1*, thereby enhancing bacterial resistance to colistin (**Figure** **2**a). For stable *mcr‐1* expression, the *mcr‐1* gene cassette, driven by its native promoter, was integrated into the *E. coli* cas locus of strain MG1655. The genome‐wide CRISPRi library was then challenged with a high dose of colistin (1 µg mL^−1^) for 4 h to selectively eliminate bacteria with minimal impact on *mcr‐1* activity. Surviving clones were selected on LB plates, and the crRNA sequences were verified by Sanger sequencing. This colistin treatment yielded over a hundred surviving clones, harboring 167 crRNAs targeting 30 different genes, many of which are involved in aminoacyl‐tRNA biosynthesis, oxidative phosphorylation, terpenoid biosynthesis, and fatty acid biosynthesis (Table S1, Supporting Information). To further investigate the effects of these candidate genes on *mcr‐1* resistance, we assessed *mcr‐1* transcription levels following CRISPR interference targeting these candidate genes. As shown in Figure S2 (Supporting Information), mRNA levels of all 30 genes were significantly inhibited, with repression ranging from 10‐ to 115‐fold. Notably, the suppression of *dinB*, *yafN*, and *yafO*, which are regulated by common regulatory elements, led to a significant upregulation of *mcr‐1* transcription, while the other genes had a negligible effect on *mcr‐1* transcript levels (Figure S3, Supporting Information). Specifically, the *yafN/O* genes are components of the *dinB* operon, which can be activated by stress‐induced mutagenesis processes, suggesting a potential regulatory role of *dinB* operon in modulating *mcr‐1* expression.

### 2.3 *dinB* Inhibits *mcr‐1* Expression and Reduces Colistin Resistance

To investigate the regulatory influence of *dinB* on *mcr‐1* expression, we compared both transcriptional and translational levels of *mcr‐1* in wild‐type MG1655 (WT) and *dinB*‐deficient (∆*dinB*) strains, both harboring *mcr‐1*. Interestingly, both mRNA and protein expression levels were markedly elevated in ∆*dinB*‐*mcr‐1* compared to WT‐*mcr‐1* (Figure 2b,c). The phosphoethanolamine catalytic activity of MCR‐1 was assessed using ITC‐PLL binding assays, which bind to Gram‐negative outer membrane in a charge‐dependent manner. The expression of *mcr‐1* reduced the negative charge on the bacterial surface, which was strengthened in the *dinB*‐deficient strain (Figure 2d). The minimum inhibitory concentration (MIC) of colistin for both ∆*dinB*‐*mcr‐1* and WT‐*mcr‐1* was 4 µg mL^−1^. However, bacterial growth curve results showed that ∆*dinB*‐*mcr‐1* exhibited a slightly higher resistance to colistin, with an IC_50_ of 2.27 µg mL^−1^ compared to an IC_50_ of 1.56 µg mL^−1^ in WT‐*mcr‐1* (Figure 2e). Moreover, following a 2 µg mL^−1^ colistin challenge, the survival rate of ∆*dinB*‐*mcr‐1* was higher than that of WT‐*mcr‐1* (Figure 2f). To further validate *dinB*’s regulatory effect on *mcr‐1* expression, we introduced complementation vectors containing the *dinB* operon and its native promoter into ∆*dinB*‐*mcr‐1*. The *mcr‐1* expression level in the complemented strain (∆*dinB*::*dinB*‐*mcr‐1*) was significantly lower than that in ∆*dinB*‐*mcr‐1* (Figure 2b,c). Correspondingly, the bacterial surface charge and colistin sensitivity were restored following *dinB* complementation (Figure 2d–f). To confirm that *dinB*’s effect on colistin sensitivity was *mcr‐1*‐dependent, we also assessed the MIC and survival rates of MG1655‐pUC19 and ∆*dinB*‐pUC19 strains. The *dinB*‐deficient strain lacking *mcr‐1* exhibited a negative outer membrane charge and colistin susceptibility similar to the WT (Figure S4, Supporting Information). These results demonstrate that *dinB* negatively regulates *mcr‐1* expression, thereby reducing colistin resistance in *E. coli*.

### 2.4 *dinB* Improves the Fitness Advantages of *mcr‐1*‐Positive *E. coli*

**Figure 3:**
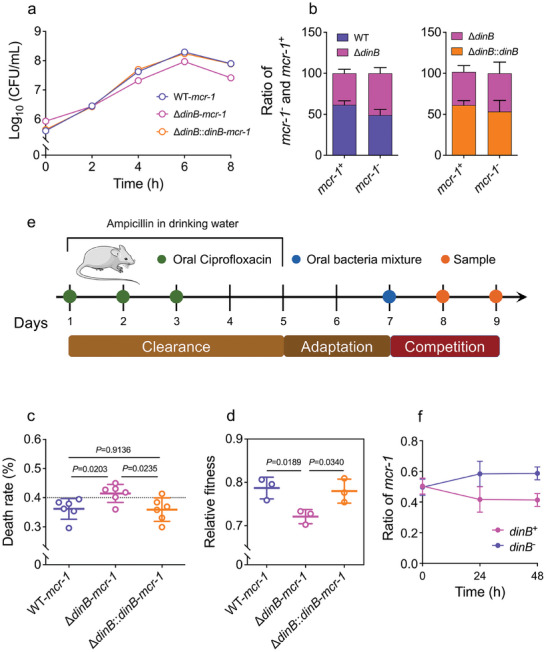
*dinB* improves the fitness advantages of *mcr‐1*‐haboring bacteria. a) Growth curves of MG1655‐*mcr‐1*, MG1655‐∆*dinB*‐*mcr‐1*, and MG1655‐∆*dinB*::*dinB*‐*mcr‐1*. b) The percentage of MG1655, MG1655‐∆*dinB*, and MG1655‐∆*dinB*::*dinB* bacteria, with or without *mcr‐1*. c) Death rate measured by SYTO9/PI live/dead bacterial double staining method. d) Relative fitness analysis of MG1655‐∆*dinB*‐*mcr‐1*, and MG1655‐∆*dinB*::*dinB*‐*mcr‐1* in competition with MG1655‐pUC20 (kanamycin resistance). e) Protocol for in vivo competition model. g) The ratio changes of *mcr‐1*‐positive *E. coli* with or without *dinB*. *p*‐values were determined using an unpaired, two‐tailed Student's *t*‐test.

As shown in Figure S5a (Supporting Information), *mcr‐1*‐negative strains maintained comparable density throughout the experiment, suggesting that *dinB* has a negligible impact on bacterial growth in the absence of *mcr‐1*. Conversely, ∆*dinB*‐*mcr‐1* exhibited significant growth retardation after 4 h, which persisted throughout the observation period (**Figure** **3**a). Furthermore, complementation with *dinB* restored the growth capacity of ∆*dinB*::*dinB*‐*mcr‐1* to levels comparable to the WT‐*mcr‐1*. Colony PCR was employed to differentiate between WT‐*mcr‐1* and ∆*dinB*‐*mcr‐1* clones as no screening markers. We found that ∆*dinB*‐*mcr‐1* clones constituted less than 40% of the population, but complementation with *dinB* restored this ratio to ≈50%, similar to that of *mcr‐1*‐negative bacteria (Figure 3b). Consistent with these findings, live‐dead cell staining results showed higher mortality rates in ∆*dinB*‐*mcr‐1* compared to WT‐*mcr‐1* or ∆*dinB*::*dinB*‐*mcr‐1* (Figure 3c). Competition assays with kanamycin‐resistant MG1655‐pUC20 further demonstrated the reduced relative fitness of ∆*dinB*‐*mcr‐1* compared to WT‐*mcr‐1* during 24‐hour co‐culture (Figure 3d). In contrast, *dinB* complementation mitigated the fitness decline associated with elevated *mcr‐1* expression due to *dinB* deficiency, with no significant difference observed between MG1655‐∆*dinB* and MG1655 in the absence of *mcr‐1* (Figure S3b, Supporting Information). After observing in vitro that *dinB* impairing the fitness advantage of *mcr‐1*‐positive bacteria, we next assessed the in vivo fitness using a murine model. WT‐*mcr‐1* and ∆*dinB*‐*mcr‐1* were mixed in a 1:1 ratio and gavaged into mice at 10^8 colony‐forming unit (CFU) mL^−1^ (Figure 3e). PCR detection of CFU in mouse feces at 24‐ and 48‐h post‐gavage showed that WT‐*mcr‐1* increased to 60% at 24 h and remained stable until 48 h, whereas ∆*dinB*‐*mcr‐1* slightly decreased after 2 days (Figure 3f). These results demonstrate that *dinB* plays a crucial role in balancing bacterial fitness and colistin resistance by regulating *mcr‐1* expression.

### 2.5 *dinB* Diminishes the Cytotoxicity of *mcr‐1*‐Positive Bacteria

**Figure 4:**
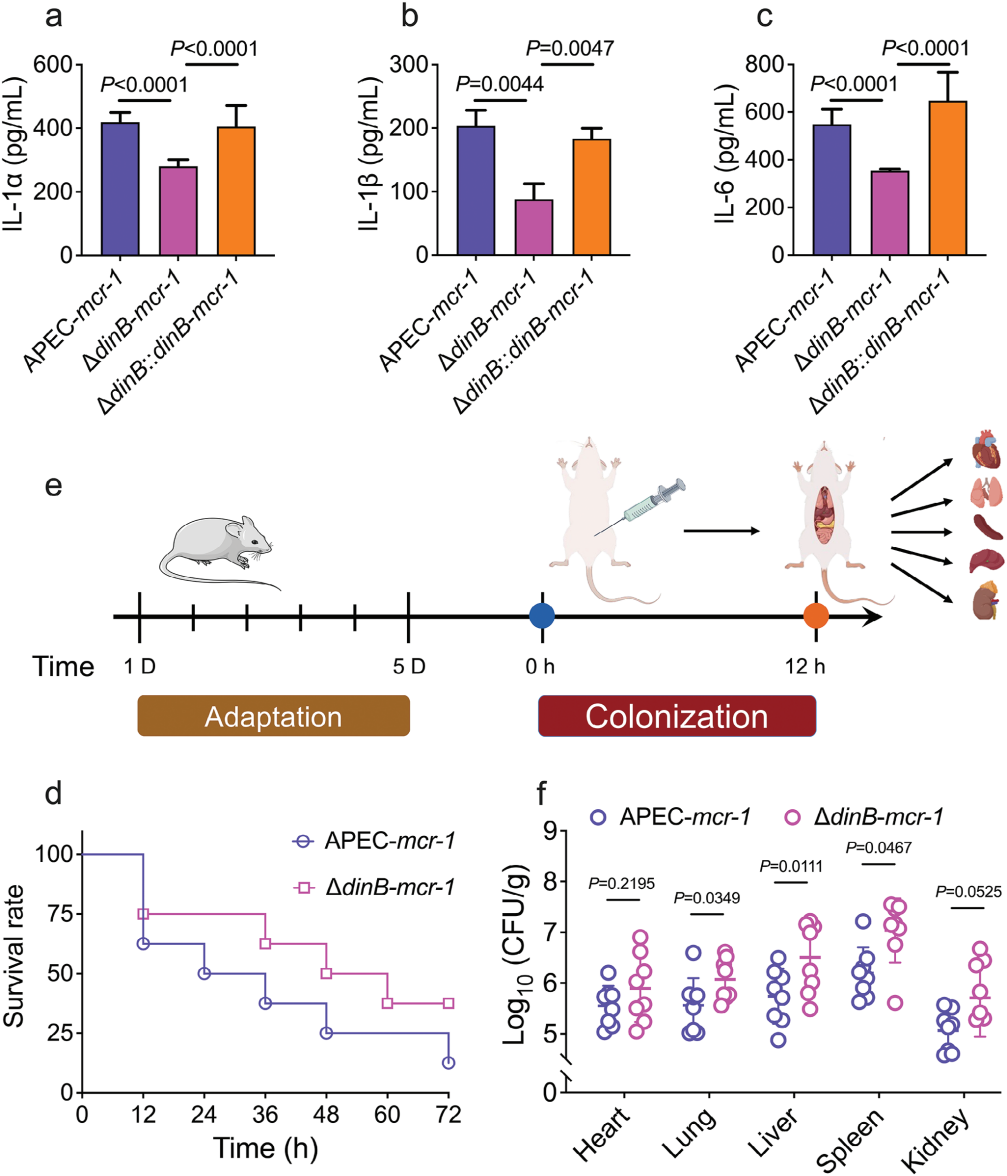
*dinB* diminishes proinflammatory response elicited by *mcr‐1*‐positive bacteria in both RAW267.4 cells and mice. RAW267.4 cells were infected at an MOI of 100 with APEC‐*mcr‐1*, APEC‐∆*dinB*‐*mcr‐1*, and APEC‐∆*dinB*::*dinB*‐*mcr‐1*. a–c) Levels of IL‐1α a), IL‐1β b), and IL‐6 c) in RAW267.4 cells were measured by ELISA. d) Survival rate of *G. mellonella* larvae over 72 h post‐infection with APEC‐*mcr‐1* and APEC‐∆*dinB*‐*mcr‐1*. e) Protocols for in vivo infection model. f) APEC‐*mcr‐1* and APEC‐∆*dinB*‐*mcr‐1* loads in the heart, lung, liver, spleen, and kidney after intraperitoneal injection. *p*‐values were determined using an unpaired, two‐tailed Student's *t*‐test.

Next, we assessed the impact of *dinB* deletion on the virulence of a clinical isolate avian pathogenic *E. coli* (APEC) harboring *mcr‐1* in both RAW267.4 cells and animal infection models. ELISA results examining the inflammatory response of RAW267.4 cells post‐infection with APEC‐*mcr‐1* and its ∆*dinB*‐*mcr‐1* strains revealed that ∆*dinB*‐*mcr‐1* infection significantly reduced the secretion of pro‐inflammatory cytokines IL‐1α, IL‐1β, and IL‐6 (**Figure** **4**a–c). Complementation with *dinB* restored pro‐inflammatory cytokine levels to those observed in APEC‐*mcr‐1*‐infected cells. Interestingly, virulence assays demonstrated that ∆*dinB*‐*mcr‐1* caused more severe pathogenicity and higher mortality in *Galleria mellonella* (*G. mellonella*) infection models (Figure 4d). To corroborate these findings, mice were intraperitoneally injected with 10^7 CFU mL^−1^ of *mcr‐1* positive bacteria with or without the *dinB* gene. Subsequently, we collected tissue samples, including heart, lung, liver, spleen, and kidney, at 12‐h post‐infection and determined their bacterial loads (Figure 4e). Notably, ∆*dinB*‐*mcr‐1* exhibited significantly higher bacterial loads than APEC‐*mcr‐1* in the lung, liver, and spleen. Additionally, the average CFUs of ∆*dinB*‐*mcr*‐1 in the heart and kidney also tended to be higher than those of APEC‐*mcr‐1* (Figure 4f). Overall, these data suggest that the decreased expression of *mcr‐1* due to *dinB* deficiency leads to increased pro‐inflammatory activity and reduced pathogenicity of *mcr‐1*‐positive *E. coli*.

### 2.6 Negative Feedback Regulation Between *mcr‐1* and *dinB*

**Figure 5:**
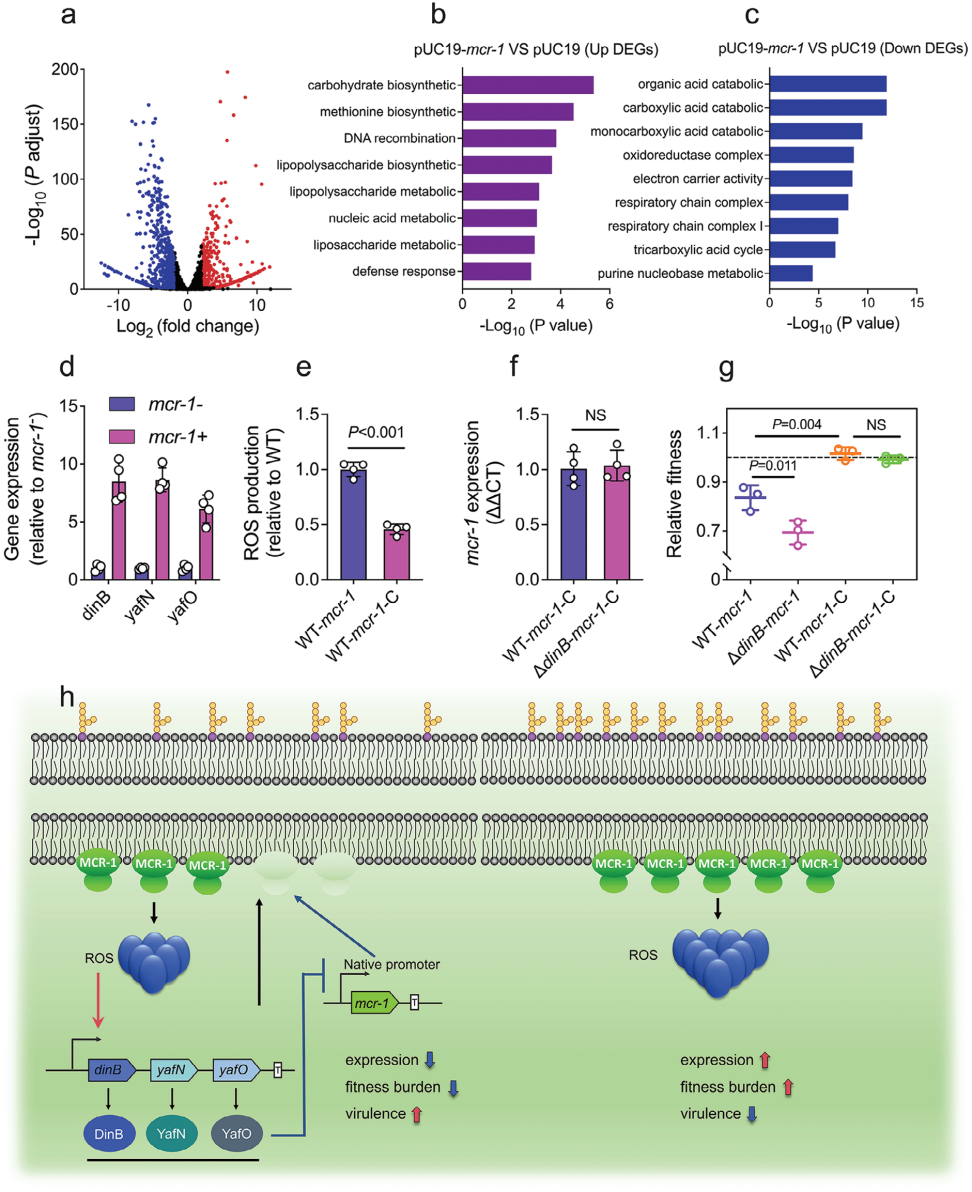
Mechanisms of the negative feedback regulation between *mcr‐1* and *dinB*. a) Differential expression analysis of MG1655‐pUC19 and MG1655‐pUC19‐*mcr‐1* by RNA‐seq. The *x*‐ and *y*‐axes represent the Log_2_ fold change and the adjusted *p* value, respectively. Genes were considered upregulated (red dots) and downregulated expressed (blue dots) when they displayed a Log_2_ fold change of more than 2 or less than ‐2, as well as an adjusted *p*‐value of less than 0.05. b and c) GO Functional enrichment of differentially upregulated b) and downregulated c) genes in MG1655‐pUC19‐*mcr‐1* compared with MG1655‐pUC19. d) Transcriptional levels of *dinB*, *yafN*, and *yafO* in MG1655‐*mcr‐1* relative to MG1655‐pUC19. e and f) Intracellular ROS level e) and transcriptional level f) of catalytic domain of *mcr‐1* (*mcr‐1*‐C). g) Relative fitness analysis of MG1655‐*mcr‐1*, MG1655‐Δ*dinB*‐*mcr‐1*, MG1655‐*mcr‐1*‐C, and MG1655‐ΔdinB‐*mcr‐1*‐C competing with MG1655‐pUC20 (kanamycin resistance). h) Schematic illustration of negative feedback regulatory mechanism between *mcr‐1* and *dinB* to maintain a balance between bacterial fitness and colistin resistance mediated by *mcr‐1*. *p*‐values were determined using an unpaired, two‐tailed Student's *t*‐test.

To better understand the relationship between *mcr‐1* and *dinB*, we performed transcriptome sequencing on *E. coli* MG1655 with or without *mcr‐1*. This analysis revealed significant alterations in gene expression in MG1655‐*mcr‐1*, with 454 genes upregulated and 514 downregulated (**Figure** **5**a). Gene Ontology (GO) analysis highlighted the functional enrichment within the transcriptome profiles following *mcr‐1* expression (Figure 5b,c). As a phosphoethanolamine (PEA) transferase, *mcr‐1* modifies lipid A in the bacterial outer membrane by attaching PEA to the N‐acetylglucosamine head.^[^ ^16^ ^]^ Consistent with this mechanism, MG1655‐*mcr‐1* exhibited significant upregulation of genes involved in lipopolysaccharide, liposaccharide, and extracellular polysaccharide metabolism (Figure 5b). Conversely, *mcr‐1* downregulated genes associated with the carboxylic acid catabolic process, electron carrier activity, monocarboxylic acid metabolism, and the tricarboxylic acid (TCA) cycle (Figure 5c). These findings confirm that *mcr‐1*‐positive bacteria (MG1655‐*mcr‐1*) undergo significant perturbations in cell cycle regulation. Interestingly, the presence of *mcr‐1* led to a substantial upregulation of *dinB* transcriptional levels, as confirmed by RT‐qPCR (Figure 5d). Furthermore, the overexpression of *mcr‐1* not only showed bacterial growth but also elevated reactive oxygen species (ROS) levels. To further explore the interaction between elevated ROS levels and *dinB* regulation, we introduced the catalytic domain of *mcr‐1* (*mcr‐1*‐C) into MG1655 and MG1655‐∆*dinB* strains. We found that intracellular ROS levels induced by *mcr‐1*‐C were significantly lower than those triggered by full‐length *mcr‐1*, resembling the negative control (Figure 5e). Importantly, the expression of *mcr‐1*‐C, which has the ability to induce ROS, was not inhibited by *dinB* (Figure 5f), and the relative fitness cost of *dinB*‐deficient bacteria carrying *mcr‐1*‐C was comparable to that of the WT (Figure 5g). These results imply that the elevation of ROS by *mcr‐1* is crucial for *dinB*‐mediated regulation of *mcr‐1* expression.

### 2.7 Construction of *mcr‐1*‐Positive *E. coli* Targeted Killing Strategy

**Figure 6:**
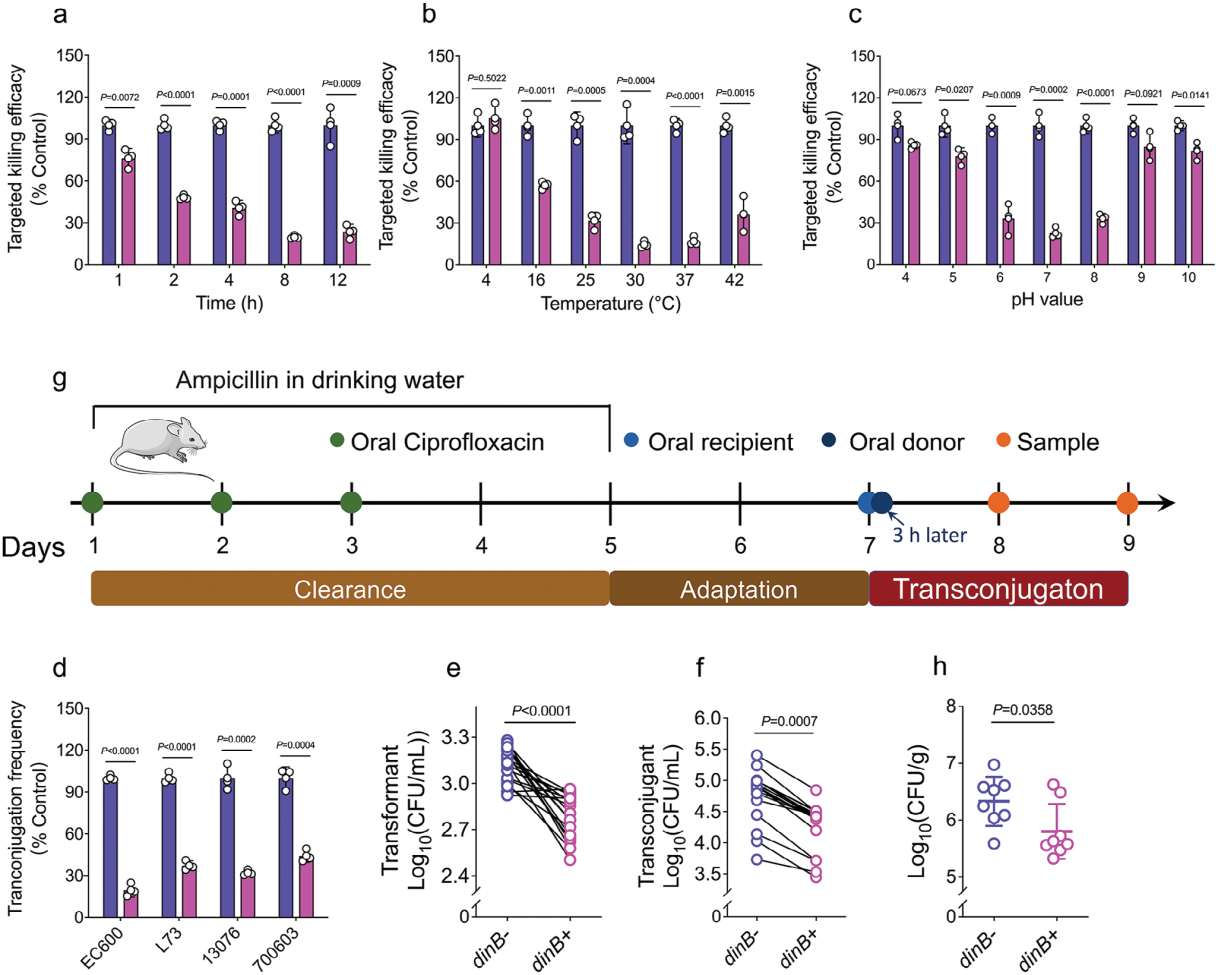
Construction of *mcr‐1*‐positive *E. coli* targeted killing system. a–c) Targeted killing efficacy of TK‐*mcr‐1* system with different mating times a), temperatures b), and pH values c). Targeted killing efficacy is measured as the ratio of survival clones between MG1655‐*mcr‐1* and MG1655‐pUC19. d) Targeted killing efficacy of TK‐*mcr‐1* system in different species including *E. coli* EC600 and L73, *S. enteritidis* ATCC13076, and *K. pneumoniae* ATCC700603. e and f) Survival transformants e) and transconjugants f) of eighteen clinical *mcr‐1*‐positive and ‐negative *E. coli* isolates treated with TK‐*mcr‐1* system with or without *dinB* promoter, respectively. g) Protocol for measuring targeted killing efficacy in vivo. h) Targeted killing efficacy measured by S17‐2 harboring the pTK‐*mcr‐1* system with or without *dinB* promoter as the donor strain, and *E. coli* B2 as the recipient strain. *p*‐values were determined using an unpaired, two‐tailed Student's *t*‐test.

Our results show that the production of *mcr‐1* triggers ROS generation, which in turn significantly upregulates *dinB* expression. Building on this phenomenon, we developed a targeted killing system for *mcr‐1*‐positive *E. coli*, utilizing a toxin protein under the control of the *dinB* promoter. During horizontal plasmid transfer via transconjugation, the basal toxin protein expression did not impede bacterial growth in the absence of *mcr‐1* induction. However, the presence of *mcr‐1* enhanced the transcriptional activity of the *dinB* promoter, increasing toxin protein expression and selectively eliminating *mcr‐1*‐positive *E. coli* (Figure S6, Supporting Information). The type II toxin CcdB, known for its DNA gyrase poisoning activity that leads to cell death, was chosen for the TK‐*mcr‐1* system. However, intact CcdB from PBAD was sufficiently toxic to kill *E. coli* hosts even without induction (Figure S7, Supporting Information). To prevent uninduced toxicity, the toxin was split using the intein, DnaE from *Nostoc punctiforme*, thus activating the split CcdB‐DnaE only in the presence of *mcr‐1*. The efficacy of the TK‐*mcr‐1* system was evaluated under various conditions, including different transconjugation times, temperatures, and pH levels. After 1 hour of transconjugation, the CFUs of *mcr‐1*‐positive *E. coli* significantly decreased compared to *mcr‐1*‐negative bacteria, with the maximum decline occurring at 8 h (**Figure** **6**a). The TK‐*mcr‐1* system was less effective at low temperatures and extreme pH levels but maintained its efficacy at temperatures above 25 °C and pH between 6 and 8 (Figure 6b,c). Additionally, the TK‐*mcr‐1* system demonstrated high killing efficiency in *Salmonella enteritidis* (*S. enteritidis*) and *K. pneumoniae* recipients (Figure 6d). We then randomly selected eighteen clinical *mcr‐1*‐positive and *mcr‐1*‐negative *E. coli* isolates and analyzed their survival rates after transformation and transconjugation with the TK‐*mcr‐1* system, with or without the *dinB* promoter. Both transformants and transconjugants of *mcr‐1*‐positive isolates exhibited significantly lower survival rates than *mcr‐1*‐negative isolates (Figure 6e,f). To assess in vivo targeted killing efficiency, a glutamine synthetase‐deficient donor strain, S17‐2 with pTK‐*mcr‐1*‐*glnA*, was gavaged 3 h after administering of *mcr‐1*‐positive or ‐negative bacteria (Figure 6g). Results indicated that S17‐2 harboring the targeted killing system significantly reduced *mcr‐1*‐positive bacterial counts (Figure 6h). These findings demonstrate that the engineered *mcr‐1*‐positive bacteria targeted killing system effectively eradicates *mcr‐1*‐positive *E. coli* in vivo.

## 3 Discussion

The emergence of plasmid‐mediated colistin resistance gene *mcr‐1* and its homologs (*mcr‐2* to *mcr‐10*) has accelerated the spread of colistin resistance, posing a significant threat to public health.^[^ ^17^ ^]^ The acquisition of *mcr‐1* drives the evolution of colistin resistance, presenting a complex evolutionary trade‐off for bacterial populations. While high‐level expression of *mcr‐1* provides robust protection against colistin, it also imposes significant physiological costs, including reduced growth rate, compromised membrane integrity, and increased cellular mortality.^[^ ^18^ ^]^ Previous research has predominantly focused on regulatory elements located on the plasmid, elucidating how plasmid‐encoded factors modulate colistin resistance and bacterial fitness costs. However, the direct interactions between *mcr‐1* and host bacterium remain poorly understood. This study systematically screened for genes involved in the regulation of *mcr‐1* expression using a genome‐wide CRISPRi crRNA library. Unlike traditional CRISPRi libraries, which require the design of multiple gRNAs for each gene and their synthesis in array‐based oligonucleotide pools,^[^ ^19^ ^]^ the genome‐wide CRISPRi crRNA library used in this study harnesses the CRISPR‐Cas adaptation machinery to incorporate sheared genomic DNA into hundreds of unique crRNAs within bacteria.^[^ ^14^, ^20^ ^]^ This approach not only identifies growth‐essential genes but also reduces costs, eliminates redundant faulty guides, and circumvents poor targeting due to limited knowledge of the molecular rules governing gRNA efficacy.^[^ ^21^ ^]^

Using this genome‐wide CRISPRi crRNA library approach, we successfully identified a novel regulatory element for *mcr‐1*, namely DNA polymerase IV DinB.^[^ ^22^ ^]^ Along with the type II toxin‐antitoxin system *yafN/O* on the *E. coli* chromosome, *dinB* forms an operon that may play a role in the feedback regulation of *mcr‐1*. Consistent with this, the deletion of *dinB* operon in *E. coli* MG1655 escalated *mcr‐1*‐mediated colistin resistance, resulting in significant fitness costs, such as impaired growth and compromised competitive ability. Further investigation revealed that *mcr‐1* activated *dinB* expression, which, in turn, downregulated *mcr‐1* expression, suggesting a negative feedback loop mechanism. In *E. coli*, *dinB*, a member of the Y family of specialized DNA polymerases, consists of three domains and is linked to an additional polymerase‐associated domain (PAD) via an extended flexible linker.^[^ ^23^ ^]^ Generally, *dinB* is upregulated as part of the SOS response, which is induced by DNA damage from UV or ionizing radiation.^[^ ^24^ ^]^

ROS are indeed endogenous agents capable of damaging DNA and inducing the mutagenic SOS response in bacteria.^[^ ^25^ ^]^ Our findings also highlighted the critical role of elevated intracellular ROS, induced by the intact *mcr‐1*, in the negative‐feedback regulatory loop. The elevated ROS levels promote overexpression of *dinB*, which subsequently suppresses the promoter activity of *mcr‐1*. When the intact *mcr‐1* was replaced with its catalytic domain, which did not induce intracellular ROS, the negative feedback regulation was disrupted. Beyond *dinB*, the expression of type II toxin‐antitoxin system YafN/O further enhances the negative feedback regulation through shared regulatory elements with *dinB*. In addition to mediating colistin resistance, studies have shown that the *mcr* gene may sever other functions.^[^ ^7^, ^26^ ^]^ *mcr‐1* confers colistin resistance by enhancing the modification of phosphoethanolamine on LPS in the bacterial cell membrane, which is closely linked to bacterial virulence.^[^ ^13a^ ^]^ For example, *mcr‐1* expression significantly bolstered the intestinal anchoring of *E. coli* by improving its fitness, which was associated with the downregulation of intestinal inflammatory markers and preservation of gut microbiota composition.^[^ ^7^ ^]^ Consistent with this, our study found that elevated *mcr‐1* expression in *dinB* knockout bacteria reduced intestinal inflammatory markers in RAW267.4 cells and enhanced colonization in various mouse tissues. While overexpression of both *dinB* and *mcr‐1* can lead to bacterial death, these proteins inhibit each other through a negative feedback loop, thereby maintaining stable bacterial growth and virulence in *mcr‐1*‐positive bacteria.^[^ ^27^ ^]^

Importantly, we developed an *mcr‐1*‐positive *E. coli* targeted killing system based on *dinB* regulation, a strategy that employs engineered *E. coli* to target and eliminate *mcr‐1*‐positive bacteria. This system utilizes the type II toxin CcdB, split by the DnaE intein and controlled by the *dinB* promoter, which is transferred into recipient strains via transconjugation.^[^ ^28^ ^]^ This approach ensures that *ccdB* expression is significantly increased in *mcr‐1*‐positive *E. coli*, confining the toxic effect to *mcr‐1*‐positive strains. In in vivo conjugation experiments, antibiotics are used to clear the native gut microbiota. This prevents the native microbiota from participating in horizontal gene transfer with recipient strains, complicating the interpretation of results. For example, non‐target strains might acquire *mcr‐1* gene, compromising the accuracy of the data. Additionally, antibiotic treatment depletes the native microbiota, reducing competition for resources and spatial niches, thereby creating a more favorable environment for conjugation. The rise of multidrug‐resistant (MDR) gram‐negative bacteria necessitates development of innovative anti‐infective strategies.^[^ ^29^ ^]^ Our study introduces a rapid and precise method to target specific antibiotic‐resistant bacterial strains, circumventing the drawbacks of broad‐spectrum antibiotics, which indiscriminately kill both pathogenic and beneficial bacteria.^[^ ^30^ ^]^ However, the potential clinical applications and off‐target effects of this targeted killing system require further investigation.

In conclusion, we characterize DNA polymerase IV *dinB* as a crucial regulator that balances *mcr‐1* expression and bacterial fitness through a novel negative‐feedback mechanism. Our findings reveal a novel regulatory element on the *E. coli* chromosome that maintains equilibrium between *mcr‐1* persistence, bacterial growth, and virulence in *mcr‐1*‐positive bacteria. By exploiting this regulatory relationship, we developed a transconjugation‐delivered *mcr‐1*‐positive *E. coli* targeted killing strategy, which effectively eliminates *mcr‐1*‐positive *E. coli* both in vitro and in vivo. Overall, our study uncovers a unique adaptive mechanism underlying *mcr‐1* persistence and facilitates the development of precision antimicrobials designed to tackle antibiotic‐resistant infections.

## 4 Experimental Section

### Bacterial Strains, Plasmids, and Growth Conditions

The bacterial strains and plasmids used or generated in this study are detailed in Tables S2 and S3 (Supporting Information), the primers employed are listed in Table S4 (Supporting Information), and the gene information is provided in Table S5 (Supporting Information). *E. coli* was cultured under static conditions on Luria–Bertani (LB) agar medium or with shaking (220 rpm) in LB broth, supplemented with appropriate antibiotics. When selection was required, the medium was supplemented with ampicillin (100 µg mL^−1^), chloramphenicol (25 µg mL^−1^), or kanamycin (50 µg mL^−1^).

### CRISPR‐Cas Adaptation‐Based CRISPRi Library Construction

The CRISPR‐Cas adaptation‐based CRISPRi library screening method was conducted as previously described.^[^ ^31^ ^]^ A single colony of *E. coli* MG1655 harboring pWJ425 was cultured overnight in 1 mL of LB broth containing ampicillin. The culture was subsequently diluted 1:100 in 100 mL fresh LB broth with 10 mM L‐arabinose to induce the expression of Cas1, Cas2, and Csn2. Bacterial growth continued until OD_620 nm_ reached 0.5 (typically after 4 h). The cells were washed twice with sterile water and 10% glycerol, respectively, at room temperature to prepare competent cells. The cells were resuspended in 1 mL of 10% glycerol and stored at −80 °C in 100 µL aliquots. Prior to electroporation, 100 µL of competent cells were incubated at room temperature for 10 min. Electroporation was performed using a Bio‐Rad MicroPulser with a single pulse of 1.8 kV (field strength of 12.5 kV cm^−1^) and a time constant of 5 ms. The bacteria were immediately resuspended in 1 mL of LB broth, transferred to a new tube, and incubated at 37 °C for 10 min with shaking. Subsequently, 500 µL of the recovered bacteria were transferred to 30 mL of LB broth containing ampicillin and incubated for another 6 h at 37 °C with shaking, allowing crRNA library adaptation during this period. To validate the coverage of the constructed crRNA library, plasmids were extracted from the 25 mL library culture using the Plasmid Mini Kit (OMEGA) following the centrifugation protocol.

Due to the low frequency of crRNA adaptation (0.1%), enrichment PCR was performed to increase the density of the adapted crRNA library for Illumina sequencing. A 100 µL reaction mixture was prepared by adding 500 ng of library plasmid as a template, 0.5 µM of forward primer (Lib01), 0.5 µM of reverse primers (an equimolar mixture of Lib02, Lib03, and Lib04), and Rapid Taq Master Mix (Vazyme). The PCR conditions were as follows: 95 °C for 120 s, followed by 30 cycles of 95 °C for 10 s, 53 °C for 10 s, and 72 °C for 20 s, followed by a final extension at 72 °C for 5 min. PCR products were visualized on a 2% agarose gel and purified using the Gel Extraction Kit (OMEGA) according to the manufacturer's instructions. The purified PCR product was sequenced using the Illumina NovaSeq PE150 platform at Suzhou GENEWIZ Inc.

### CRISPRi Library Screening

Expression of *mcr‐1* was tightly regulated, as its overexpression impairs not only bacterial growth, viability, and competition but also cellular membranes and cytoplasmic components. To identify genes involved in the negative regulation of *mcr‐1*‐induced colistin resistance, a CRISPR‐Cas adaptation‐based CRISPRi library for *E. coli* MG1655/*mcr‐1* as previously described was constructed. Subsequently, the library was exposed to a high concentration of colistin to isolate surviving clones. Specifically, the library construction was based on a CRISPR‐Cas adaptation system, wherein *E. coli* MG1655/*mcr‐1* competent cells were electroporated to integrate genomic DNA into a CRISPR array, thereby generating a pool of crRNAs. The CRISPRi library for *E. coli* MG1655/*mcr‐1* was thawed and diluted 1:100 into 5 mL of LB broth. One milliliter of the library was plated onto 12 cm × 12 cm square LB agar plates supplemented with 1 µg mL^−1^ colistin. The plates were incubated at 37 °C for 12 h, after which all surviving clones were selected into LB broth containing ampicillin and subjected to Sanger sequencing to determine the crRNA sequences.

### RNA Extraction and RT‐qPCR Analysis

Bacteria were cultured in LB broth at 37 °C overnight, then inoculated at a 1:100 dilution in 3 mL of fresh LB medium and incubated with shaking at 200 rpm for ≈4 h at 37 °C until the logarithmic growth phase was reached. Total bacterial RNA was extracted using the Bacterial RNA Extraction Kit (Vazyme) according to the manufacturer's instructions. One microgram of extracted RNA from each sample was used as a template for cDNA synthesis using the HiScript III RT SuperMix (Vazyme). Quantitative real‐time PCR (qRT‐PCR) analysis was performed using 50 ng of cDNA as the template, with 16S rRNA serving as the reference internal control. Targets of ≈150 bp were amplified using the ChamQ SYBR Color qPCR Master Mix Kit (Vazyme) and primers listed in Table S3 (Supporting Information). Relative transcript levels were analyzed using the relative quantitative (2^−ΔΔCt^) method, as previously described.^[^ ^32^ ^]^

### RNA Sequencing

Cultures of MG1655‐pUC19 and MG1655‐*mcr‐1* in LB broth during the logarithmic growth phase were selected for RNA‐Seq analysis. Total RNA was extracted using TRIzol reagent (Invitrogen) following the manufacturer's instructions, and genomic DNA was removed using DNase I (Takara). RNA quality was assessed using the 2100 Bioanalyzer (Agilent) and quantified with the ND‐2000 instrument (NanoDrop). The transcriptome sequencing library was prepared with the TruSeq RNA sample preparation kit (Illumina) using 2 µg of total RNA. Following quantification with a TBS380 instrument, the library was sequenced on the Illumina HiSeq X Ten system (2 × 150 bp read length) and analyzed using the Illumina Genome Analyzer (GA) pipeline (v.1.6), producing 150‐bp paired‐end reads. These reads were aligned to the *E. coli* K‐12 strain (NCBI reference sequence NC_000913.3).

### Western Blot Analysis

Western blot analysis was performed following established protocols. Briefly, overnight bacterial cultures were diluted 1:100 into 3 mL of fresh LB medium and incubated at 37 °C with shaking at 200 rpm for ≈4 h to reach the logarithmic growth phase. Five hundred microliters of each sample were transferred to a new tube, pelleted by centrifugation at 5000 rpm for 5 min, and resuspended in 20 µL of SDS‐PAGE sample buffer (Thermo Scientific). Samples were lysed by sonication and heated at 95 °C for 5 min, followed by centrifugation at 8000 rpm for 5 min. The supernatant was separated by 10% sodium dodecyl sulfate‐polyacrylamide gel electrophoresis (SDS‐PAGE) for 90 min at 100 V and transferred to a PVDF membrane for 120 min at 200 mV. The membrane was blocked with NcmBlot Blocking Buffer (Ncmbio) for 60 min. Immunoblotting was performed using an anti‐*mcr‐1* mouse polyclonal antibody as the primary antibody and an HRP‐conjugated goat anti‐mouse antiserum (Jackson Immuno Research Inc.) as the secondary antibody for 1 h at room temperature, according to previously described methods.^[^ ^33^ ^]^ Bound antibodies were detected using an ECL reagent (Thermo Scientific) and visualized using a ChemiDoc XRS+ (Bio‐Rad). Relative band intensities were quantified using ImageJ version 1.49 software (NIH).

### Bacterial Surface Charge Measurements

Overnight bacterial cultures were diluted 1:100 into 3 mL of fresh LB medium and incubated at 37 °C with shaking at 200 rpm for ≈4 h to reach the logarithmic growth phase. The bacteria were then washed twice and diluted 1:5 in saline solution to a final OD_620 nm_ of 0.1. FITC‐labeled poly‐L‐lysine (FITC‐PLL) solution was diluted 1:2000 into each sample to a final concentration of 2 µg mL^−1^. The fluorescence (Ex‐500 nm/Em‐530 nm) of the cultures was measured using the Multiscan Spectrum (Tecan) after a 15‐minute incubation at room temperature. The relative surface charge was compared with PBS controls to determine the proportion of bacteria‐bound dye.

### Determination of Colistin Susceptibility

Colistin susceptibility was assessed following the Clinical Laboratory Standards Institute (CLSI) guidelines. Log‐phase cultures of all strains were diluted 1:1000 and plated in colistin with twofold serial dilutions ranging from 0.0078 to 8 mg mL^−1^ in a 96‐well plate. The plates were incubated at 37 °C for 18 h in the dark. Measurements were performed in triplicate, including positive and negative controls for each MIC determination. After incubation, the cultures were gently mixed, and the absorbance at OD_620 nm_ was measured using a microplate reader (Bio‐Rad). MIC was defined as the lowest concentration of colistin at which OD_620 nm_ less than 0.1. *IC_50_* (the concentration of colistin causing 50% maximal bacterial inhibition) was calculated using the log (inhibitor) versus response–Variable slope (four parameters) model in GraphPad Prism version 10.3.0 software (San Diego), based on the equation:

(1) $$Y=Bottom+\left(Top-Bottom\right)/\left(1+10^{\left(\left(log_{2}IC_{50}-X\right)*HillSlope\right)}\right)$$

### Time‐Kill Assay

Overnight cultures were diluted 1:1000 into fresh LB medium and incubated for 4 h at 37 °C with shaking at 200 rpm. The bacteria were then collected and resuspended in MH Broth at 10^7 CFU mL^−1^, and treated with 4 µg mL^−1^ colistin in a shaking incubator at 37 °C. Samples were collected at 0, 1, 2, 4, 6, and 12 h, and 10‐fold serial dilutions were performed to quantify CFU at each time point.

### Growth Curve

To explore the fitness impact of *mcr‐1* overexpression due to *dinB* deficiency, the growth of MG1655 and MG1655‐∆*dinB*, both with and without *mcr‐1*, was initially examined. Log‐phase cultures were adjusted to an optical density of 0.5 at OD_620 nm_ and then diluted 1:1000 in fresh LB broth. The cultures were incubated at 37 °C with shaking at 200 rpm for 12 h. Samples were collected at 4, 6, 8, and 12 h, serially diluted in sterile PBS, and spot‐plated onto LB plates to measure CFU at each time point.

### Competition Experiments In Vitro

Overnight cultures grown from a single colony were adjusted to an optical density of 0.5 at OD_620 nm_ and mixed in equal volumes. The mixtures were diluted 1:1000 into fresh LB broth and grown for 24 h. At 0 and 24 h, the cultures were serially diluted and plated onto LB agar plates containing different antibiotics for screening. To further assess the effect of *dinB* deletion on *mcr‐1*‐positive bacterial growth and fitness in vitro, equal quantities of WT‐*mcr‐1* and ∆*dinB*‐*mcr‐1* were mixed and incubated for 24 h. As WT and ∆*dinB* cannot be distinguished by specific antibiotics, these two competitor strains were differentiated by colony PCR with primers W1205/W1206. The formula RF = ln(NfA/NiA)/ln(NfB/NiB) was used to calculate the relative competition factor between the two strains, where RF represents the relative fitness of strain A compared to strain B. NiA and NfA represent the numbers of strain A at the beginning and end of the competition, while NiB and NfB represent the numbers of strain B at the beginning and end of the competition, respectively.

### RAW264.7 Macrophage Infection

Macrophage RAW264.7 cells from mice were cultured in DMEM with 10% FBS (Sigma‐Aldrich) in a humidified incubator with 5% CO_2_ at 37 °C. The cells were plated in 6‐well clusters (35 mm diameter) to achieve 60–80% confluency. To evaluate whether the eukaryotic cellular pathogenicity is associated with increased *mcr‐1* expression, the inflammatory status of RAW264.7 cells after infection with WT‐*mcr‐1* and ∆*dinB*‐*mcr‐1* strains was assessed using ELISA. Before adding bacteria, the medium was replaced with DMEM without FBS. The RAW 264.7 cells treated with PBS served as the negative control. The wells were incubated at 37 °C for 12 h. Subsequently, samples were collected and stored at −20 °C. The production of IL‐1α, IL‐1β, and IL‐6 was measured using an ELISA kit (R&D Systems), and cytokine concentrations were calculated according to the manufacturer's instructions.

### Ethical Statement

This study was performed according to the relevant guidelines of Jiangsu Laboratory Animal Welfare and Ethical of Jiangsu Administrative Committee of Laboratory Animals (SYXK‐2022‐0044). All animal experiments were approved by the Animal Care Committee of Yangzhou University.

### *mcr‐1‐*Positive *E. coli* Targeted Killing

Conjugation assays utilized S17‐2 harboring pTK‐*mcr‐1* as the donor strain and *E. coli* MG1655 transformed with pUC19 or pUC19‐*mcr‐1* as recipient strains. Overnight‐grown donor and recipient strains were diluted 1:100 in fresh medium and incubated until reaching an OD600 of 0.5. After washing with PBS, the recipient strains were resuspended in PBS and diluted to various concentrations. Donor and recipient strains were then mixed at a 1:0.1 ratio, and 50 µL of the mixture was applied on GN‐6 membranes (Pall) placed on LB agar plates. These mixtures were subjected to different incubation conditions, including varying transconjugation durations (1, 2, 4, 8, and 12 h), temperatures (4, 16, 25, 30, 37, and 42 °C), and pH levels (4–10). The GN‐6 membranes were subsequently removed and transferred into 1 mL of sterile PBS, thoroughly vortexed, serially diluted in PBS, and plated on LB agar supplemented with different antibiotics. 100 µg mL^−1^ ampicillin with or without 50 µg mL^−1^ kanamycin was used to selectively isolate the recipients and transconjugants.

The in vivo *mcr‐1*‐positive *E. coli* targeted killing procedure was as follows: Female BALB/c mice (6–8 weeks old) were obtained from Yangzhou University's Comparative Medicine Centre (Jiangsu, China) and acclimatized for a week before infection. To clear gut microbiota, 2 mg of ciprofloxacin dissolved in 100 µL sterile dH_2_O was administered by oral gavage for three consecutive days. After each ciprofloxacin treatment, mice were transferred to new cages to minimize intestinal recolonization from the environment. Additionally, drinking water containing 1 g L^−1^ streptomycin, 0.5 g L^−1^ ampicillin, 1 g L^−1^ gentamicin, and 0.5 g L^−1^ vancomycin was provided for five days. In vivo conjugation assays involved *E. coli* MG1655 with or without *mcr‐1* and of S17‐2 transformed with pTK‐*mcr‐1* with or without the *dinB* promoter, after drinking fresh water without antibiotics for two days. Female ICR mice (*n* = 8 per group) received oral administration of 10^9 CFU of the donor strain 4 h after receiving 10^8 CFU of the recipient strain. Fecal samples were collected, weighed, diluted in PBS, and plated onto LB agar with appropriate antibiotics to count CFU.

## Conflict of Interest

The authors declare no conflict of interest.

## Author Contributions

H.Z. and X.X. contributed equally to this work as co‐first authors. Y.L. and Z.W. designed and supervised the project; H.Z. performed experiments, analyzed data, and drafted the manuscript; X.X. helped to analyze data; Y.Z., C.W., B.C., X.J., L.G., and J.W. helped to perform experiments; Y.L. and H.Z. wrote and revised the manuscript. All of the authors read and approved the final manuscript.

## Supporting information



Supporting Information

## Data Availability

The data that support the findings of this study are available from the corresponding author upon reasonable request.
