# Supplementary material for: DNA Polymerase IV dinB Favors the Adaptive Fitness of mcr‐carrying Bacteria Through a Negative Feedback Regulatory Mechanism
Source: Adv Sci (Weinh). 2025 Jan 31;12(12):2411994. doi: 10.1002/advs.202411994 (PMC11948064; doi:10.1002/advs.202411994)
Supplement: Supplementary file 1 — Supporting Information [file ADVS-12-2411994-s001.docx]

**Supplementary Information**


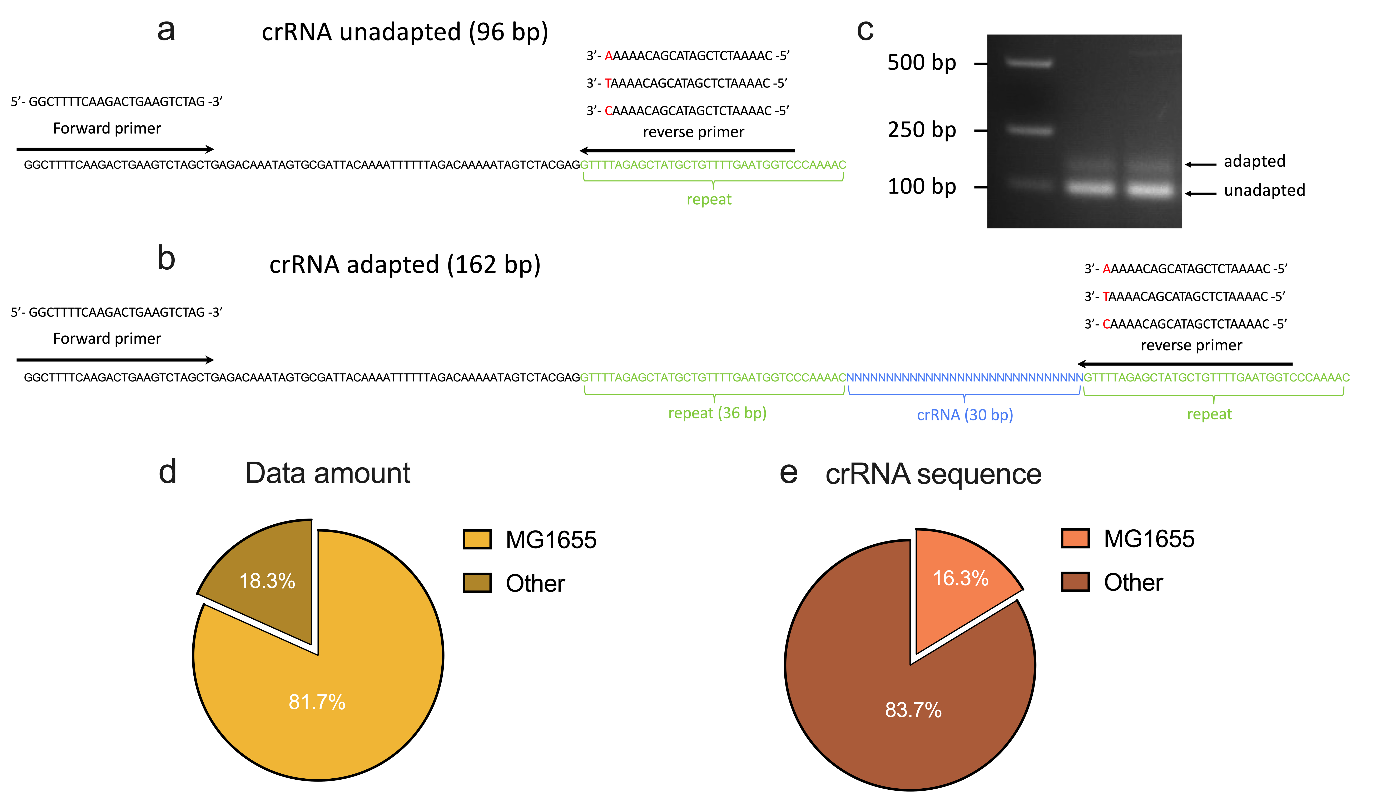


**Supplementary Figure S1. Generation of genome-wide CRISPRi crRNA library, related to Figure 1.**

(a and b) Sequences of the unadapted **(a)** and adapted **(b)** CRISPR array, forward primer (Lib01) and reverse enrichment primers (equimolar mixture of Lib02, Lib03, and Lib04). **(c)** Adapted crRNA locations amplified by enrichment primers. The PCR products were detected on 2% agarose gel. The upper (162 bp) and lower (96 bp) bands corresponded to the adapted and unadapted CRISPR arrays, respectively. The data amount **(d)** and crRNA sequences **(e)** revealed by deep sequencing.


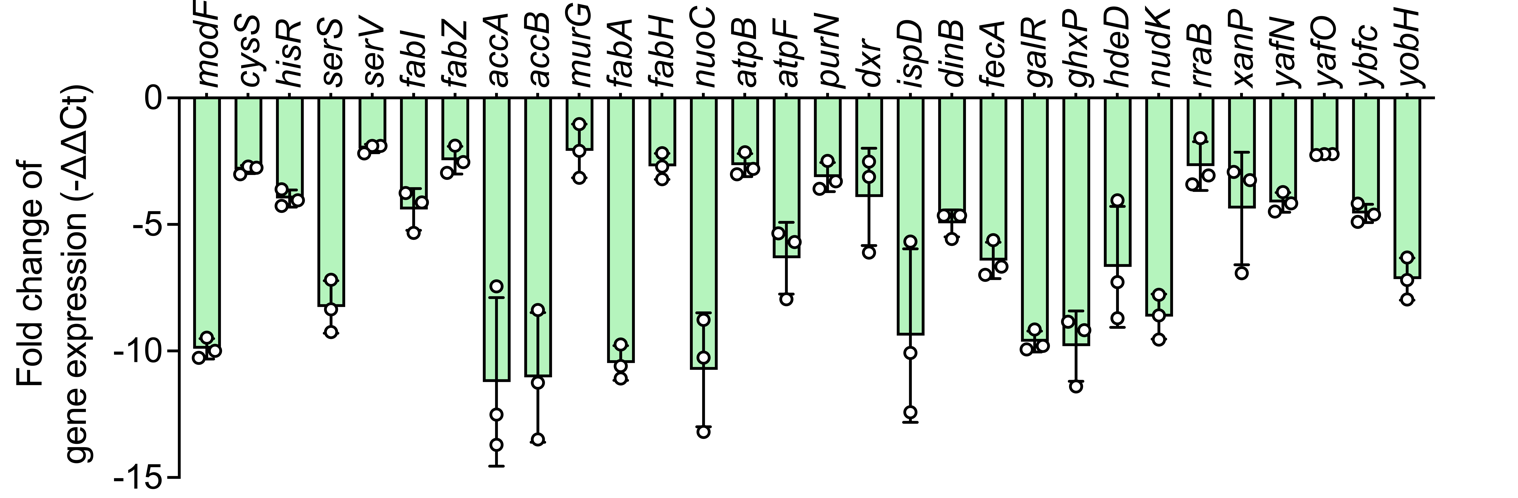


**Supplementary Figure S2.** The mRNA expression levels of 30 candidate genes inhibited by CRISPRi method.


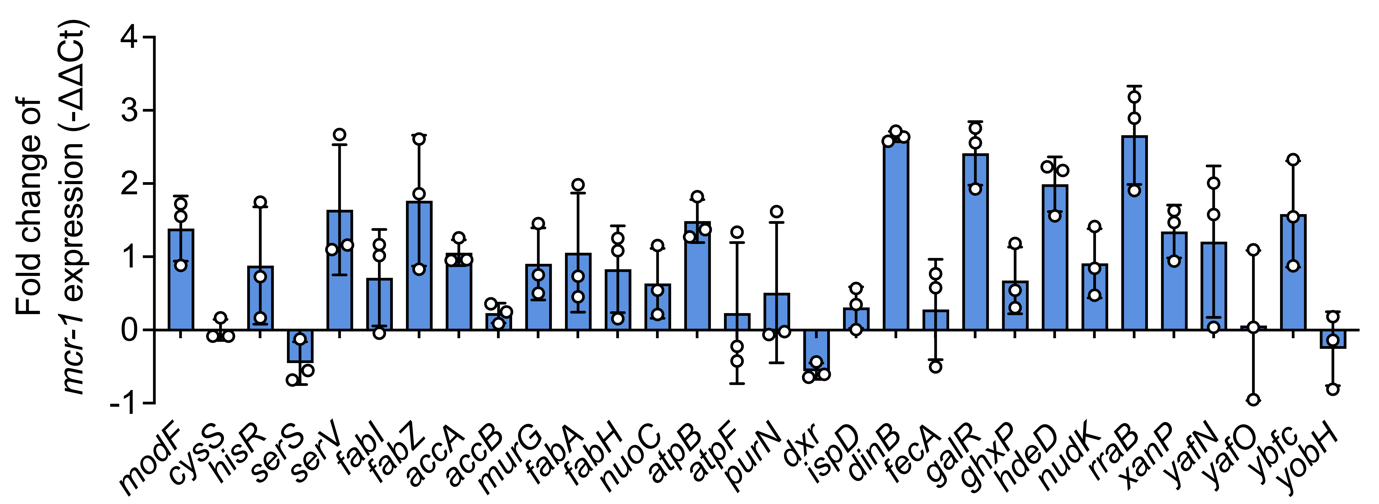


**Supplementary Figure S3.** The mRNA expression levels of *mcr-1* after the candidate gene expression is silenced by CRISPRi method.


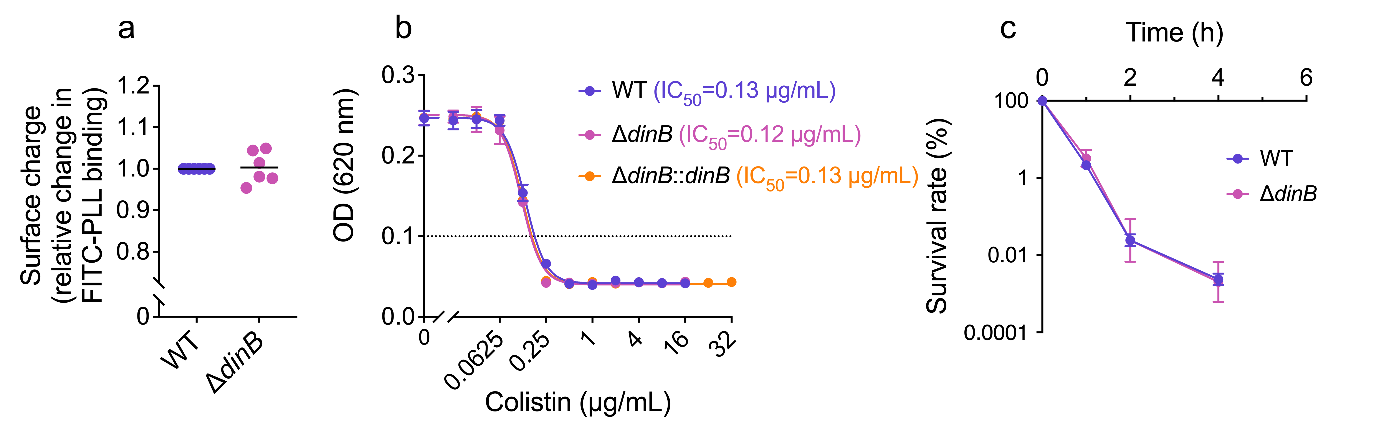


**Supplementary Figure S4.** The relative bacterial surface negative charge **(a)**, absorbance at OD_620 nm_ with increasing concentrations of colistin **(b)**, and survival rate upon 8 μg/mL colistin treatment **(c)** of MG1655 and MG1655-∆*dinB*.


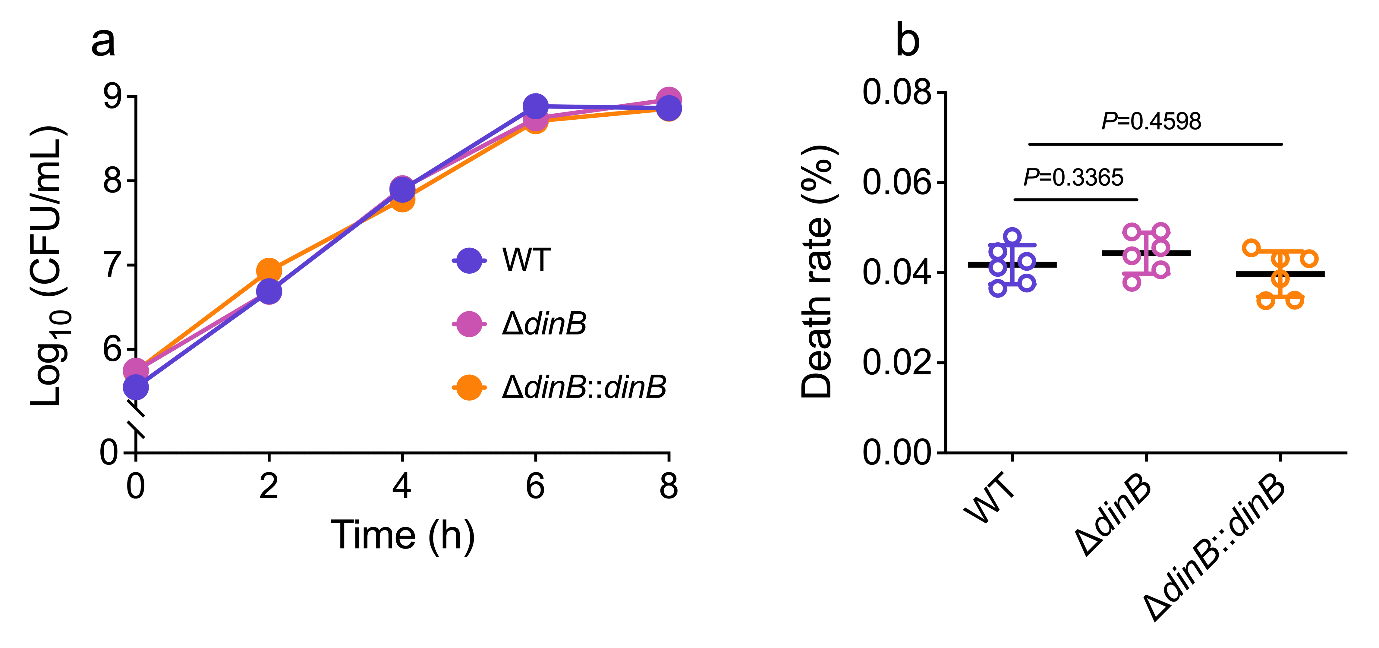


**Supplementary Figure S5.** Growth curves **(a)** and death rates **(b)** of MG1655, MG1655-∆*dinB* and MG1655-∆*dinB*::*dinB*.


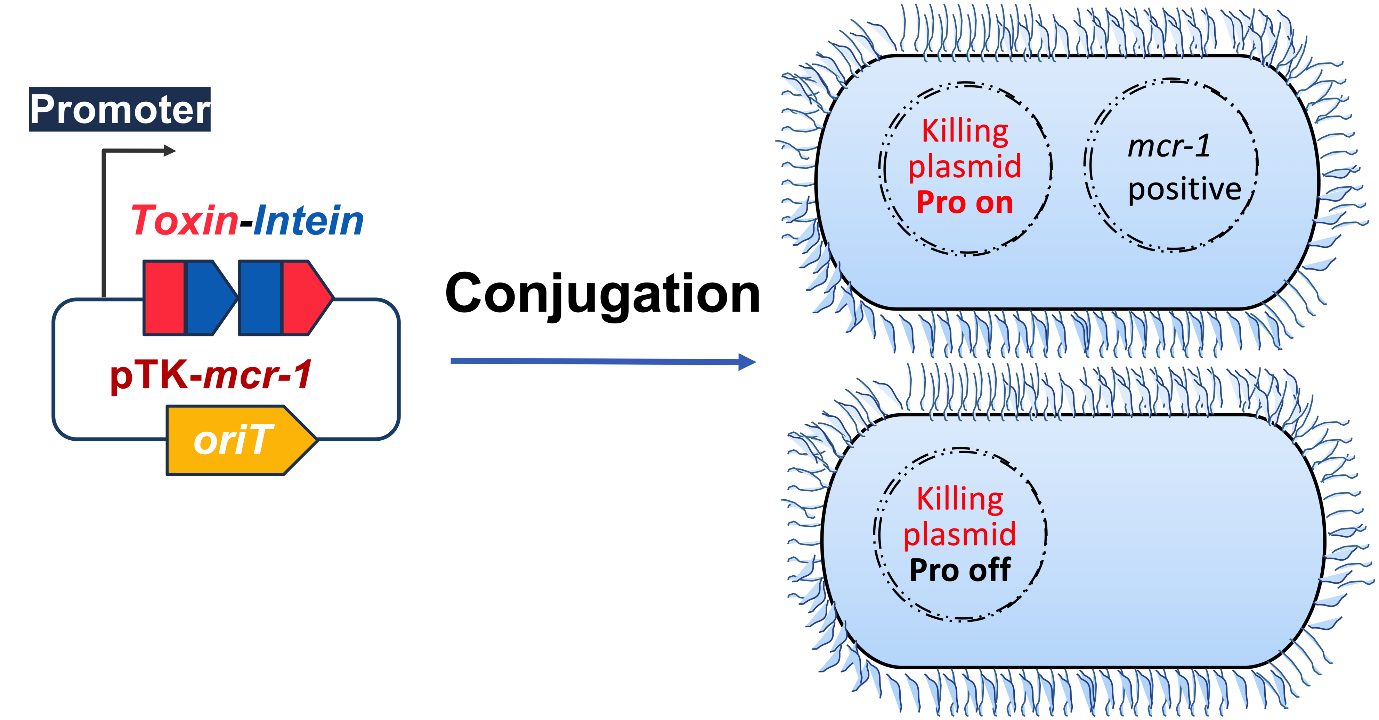


**Supplementary Figure S6.** The schematic illustration for *mcr-1*-positive *E. coli* target killing system.


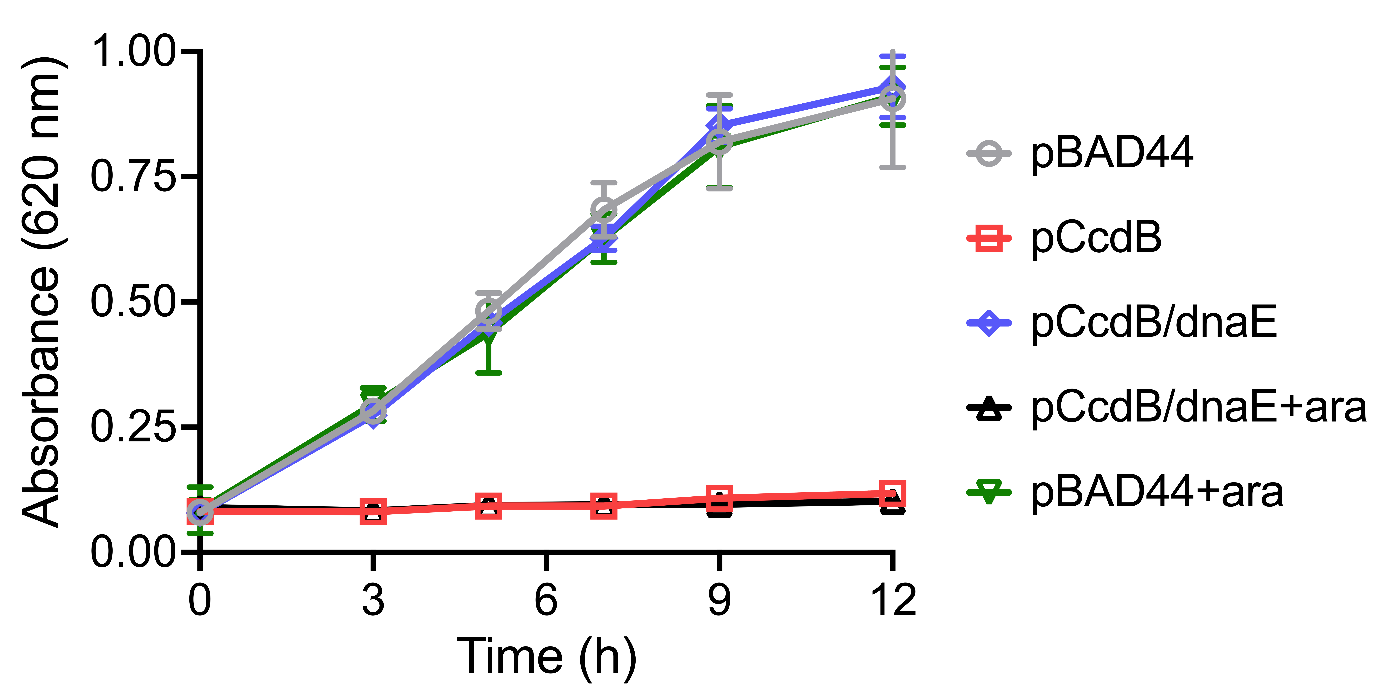


**Supplementary Figure S7.** The toxicity of different types of CcdB against *E. coli*.

**Supplementary Table S1. Survival clones after being treated with high concentrations of colistin.**

| Genes | ID | Description | KEGG pathway |
| --- | --- | --- | --- |
| *modF* | b0760 | ABC family protein ModF | ABC transporters |
| *cysS* | b0526 | cysteine-tRNA ligase | Aminoacyl-tRNA biosynthesis |
| *hisR* | b3797 | tRNA-His | Aminoacyl-tRNA biosynthesis |
| *serS* | b0893 | serine-tRNA ligase | Aminoacyl-tRNA biosynthesis |
| *serV* | b2695 | tRNA-Ser | Aminoacyl-tRNA biosynthesis |
| *fabI* | b1288 | enoyl-[acyl-carrier-protein] reductase | Biotin metabolism; Fatty acid biosynthesis |
| *fabZ* | b0180 | 3-hydroxy-acyl-[acyl-carrier-protein] dehydratase | Biotin metabolism; Fatty acid biosynthesis |
| *accA* | b0185 | acetyl-CoA carboxyltransferase subunit alpha | Fatty acid biosynthesis; Pyruvate metabolism |
| *accB* | b3255 | biotin carboxyl carrier protein | Fatty acid biosynthesis; Pyruvate metabolism |
| *murG* | b0090 | N-acetylglucosaminyl transferase | Cell cycle - Caulobacter |
| *fabA* | b0954 | beta-hydroxyacyl-acyl carrier protein dehydratase/isomerase | Fatty acid biosynthesis |
| *fabH* | b1091 | beta-ketoacyl-[acyl carrier protein] synthase III | Fatty acid biosynthesis |
| *nuoC* | b2286 | NADH:quinone oxidoreductase subunit CD | Oxidative phosphorylation |
| *atpB* | b3738 | ATP synthase Fo complex subunit a | Oxidative phosphorylation |
| *atpF* | b3736 | ATP synthase Fo complex subunit b | Oxidative phosphorylation |
| *purN* | b2500 | phosphoribosylglycinamide formyltransferase 1 | Purine metabolism; One carbon pool by folate |
| *dxr* | b0173 | 1-deoxy-D-xylulose 5-phosphate reductoisomerase | Terpenoid backbone biosynthesis |
| *ispD* | b2747 | 2-C-methyl-D-erythritol 4-phosphate cytidylyltransferase | Terpenoid backbone biosynthesis |
| *dinB* | b0231 | DNA polymerase IV |  |
| *fecA* | b4291 | ferric citrate outer membrane transporter |  |
| *galR* | b2837 | DNA-binding transcriptional dual regulator GalR |  |
| *ghxP* | b4064 | guanine/hypoxanthine transporter GhxP |  |
| *hdeD* | b3511 | acid-resistance membrane protein |  |
| *nudK* | b2467 | GDP-mannose hydrolase |  |
| *rraB* | b4255 | ribonuclease E inhibitor protein B |  |
| *xanP* | b3654 | xanthine:H(+) symporter XanP |  |
| *yafN* | b0232 | antitoxin YafN |  |
| *yafO* | b0233 | ribosome-dependent mRNA interferase toxin YafO |  |
| *ybfc* | b0704 | uncharacterized protein YbfC |  |
| *yobH* | b4536 | protein YobH |  |

**Supplementary Table S2. Strains used in this study.**

| Strains | Description | Sources/References |
| --- | --- | --- |
| DH5α | *F^-^ φ80 lacZ*Δ*M15* Δ*(lacZYA-argF) U169 recA1 endA1 hsdR17(rk^-^, mk^+^) phoA, supE44 thi-1 gyra96* | Laboratory stock |
| MG1655 | *F^-^  ilvG rfb-50 rph-1* | Laboratory stock |
| APEC TW XM | Clinical isolated avian pathogenic E. coli TW XM, O2:K1:H7 | [1] |
| S17-1 | *RP4-2(Km::Tn7,Tc::Mu-1) pro-82 LAMpir recA1 endA1 thiE1 hsdR17 creC510* | Laboratory stock |
| *x7213* | thi-1 thr-1 leuB6 fhuA21 lacY1 glnV44 asdA4 recA1 RP4 2-Tc::Mu pir; Km^r^ | Laboratory stock |
| MG1655-∆*dinB* | MG1655 *∆dinB ∆yafN ∆yafO* | This study |
| APEC-∆*dinB* | APEC TW XM *∆dinB ∆yafN ∆yafO* | This study |
| S17-2 | *E. coli* S17-1 Δ*glnA* | [2] |
| EC600 | *E. coli* (Rif^R^) | Laboratory stock |
| L73 | *E. coli* with *mcr-1* integrated into the genome | [3] |
| 700603 | *K. pneumoniae* | ATCC 700603 |
| 13076 | *Salmonella enterica* subsp. *enterica* | ATCC 13076 |

**Supplementary Table S3. Plasmids used in this study.**

| Plasmids | Description | Sources/References |
| --- | --- | --- |
| pUC19 | ColE1, (Amp^R^) | Laboratory stock |
| pUC20 | ColE1, (Kan^R^) | This study |
| pRE112 | Widely used gene-knocked suicide vector with oriT RP4 and R6K γ ori, (Cm^R^) | Laboratory stock |
| pBAD44 | pCS101, (Kan^R^) | Laboratory stock |
| pWJ425 | tracr, dcas9-hyper, repreat, araBAD promoter, cas1, cas2, csn2, p15A, (Amp^R^) | This study |
| pRE112-Δ*dinB* | pRE112 containing the homologous arms of *dinB*/*yafN*/*yafO*, (Cm^R^) | This study |
| pUC19-*mcr-1* | *mcr-1* under control of its native promoter, ColE1, (Amp^R^) | [4] |
| pBAD44-*dinB* | *dinB*, *yafN* and *yafO* under control of its native promoter, pCS101, (Sm^R^) | This study |
| pccdB | *ccdB* under control of arabinose promoter in pBAD44 | This study |
| pccdB/dnaE | *dnaE* splited *ccdB* under control of arabinose promoter in pBAD44 | This study |
| pTK-*mcr-1* | *dnaE* splited *ccdB* under control of *dinB* promoter in pBAD44 | This study |
| psgRNA | sgRNA expression vector, ColE1, (Kan^R^) | This study |
| psgRNA-*modF* | psgRNA with sgRNA targeted *modF* | This study |
| psgRNA-*cysS* | psgRNA with sgRNA targeted *cysS* | This study |
| psgRNA-*hisR* | psgRNA with sgRNA targeted *hisR* | This study |
| psgRNA-*serS* | psgRNA with sgRNA targeted *serS* | This study |
| psgRNA-*serV* | psgRNA with sgRNA targeted *serV* | This study |
| psgRNA-*fabI* | psgRNA with sgRNA targeted *fabI* | This study |
| psgRNA-*fabZ* | psgRNA with sgRNA targeted *fabZ* | This study |
| psgRNA-*accA* | psgRNA with sgRNA targeted *accA* | This study |
| psgRNA-*accB* | psgRNA with sgRNA targeted *accB* | This study |
| psgRNA-*murG* | psgRNA with sgRNA targeted *murG* | This study |
| psgRNA-*fabA* | psgRNA with sgRNA targeted *fabA* | This study |
| psgRNA-*fabH* | psgRNA with sgRNA targeted *fabH* | This study |
| psgRNA-*nuoC* | psgRNA with sgRNA targeted *nuoC* | This study |
| psgRNA-*atpB* | psgRNA with sgRNA targeted *atpB* | This study |
| psgRNA-*atpF* | psgRNA with sgRNA targeted *atpF* | This study |
| psgRNA-*purN* | psgRNA with sgRNA targeted *purN* | This study |
| psgRNA-*dxr* | psgRNA with sgRNA targeted *dxr* | This study |
| psgRNA-*ispD* | psgRNA with sgRNA targeted *ispD* | This study |
| psgRNA-*dinB* | psgRNA with sgRNA targeted *dinB* | This study |
| psgRNA-*fecA* | psgRNA with sgRNA targeted *fecA* | This study |
| psgRNA-*galR* | psgRNA with sgRNA targeted *galR* | This study |
| psgRNA-*ghxP* | psgRNA with sgRNA targeted *ghxP* | This study |
| psgRNA-*hdeD* | psgRNA with sgRNA targeted *hdeD* | This study |
| psgRNA-*nudK* | psgRNA with sgRNA targeted *nudK* | This study |
| psgRNA-*rraB* | psgRNA with sgRNA targeted *rraB* | This study |
| psgRNA-*xanP* | psgRNA with sgRNA targeted *xanP* | This study |
| psgRNA-*yafN* | psgRNA with sgRNA targeted *yafN* | This study |
| psgRNA-*yafO* | psgRNA with sgRNA targeted *yafO* | This study |
| psgRNA-*ybfC* | psgRNA with sgRNA targeted *ybfC* | This study |
| psgRNA-*yobH* | psgRNA with sgRNA targeted *yobH* | This study |

**Supplementary Table S4. Oligonucleotides used in this study.**

| Primers | Sequences |
| --- | --- |
| sgRNA_F | AGACCGAGCGCCTGGGTCGCGATCGCGGACCCGTAAAGTGATAA |
| sgRNA_R | GTCGTTCGACTGCGGCGAGGCGATCCCGGGATTAAGTTCTGTGCTAGG |
| q*mcr-1*_F | GAACGCTACAAAGAACTGATT |
| q*mcr-1*_R | TAGCCTCTTCAATGCTGTTA |
| q*dinB*_F | CCCACATCTCACCTTGCTTC |
| q*dinB*_R | TCGGTGACATCGAGATAAG |
| q*yafN*_F | GGTTGCGGTTCTTTCTAA |
| q*yafN*_R | AATCTTGCAGCACTTGGAC |
| q*yafO*_F | GGTCGCGATGCACTCTAC |
| q*yafO*_R | TACCAAATGCGCTTCGTC |
| 16S rRNA_F | GTTAATAACTTTGCTCATTGA |
| 16S rRNA_R | ACCAGGGTATCTAATCCTGTT |
| *dinB*-up_F | TCCCAAGCTTCTTCTAGAGTTTGAACGCGGCAGCGC |
| *dinB*-up_R | ATTCATAATATAAATTCAATGAATGATTTTACGCAT |
| *dinB*-down_F | ATGCGTAAAATCATTCATTGAATTTATATTATGAAT |
| *dinB*-down_R | ACTGCATGAATTCCCGGGTACCAGGCGGGCGTTATT |
| *dinB*-112_F | GCGCTGCCGCGTTCAAACTCTAGAAGAAGCTTGGGA |
| *dinB*-112_R | AATAACGCCCGCCTGGTACCCGGGAATTCATGCAGT |
| *dinB*_F | GTGCCGGAAGAGACGGAG |
| *dinB*_R | TCCGGGGTGTAAATCTGG |
| pUC19_F | ACTCTTCCTTTTTCAATATTA |
| pUC19_R | CTGTCAGACCAAGTTTACTCA |
| *kan*_F | TATTGAAAAAGGAAGAGTATGAGCCATATTCAACGGG |
| *kan*_R | TAAACTTGGTCTGACAGTTAGAAAAACTCATCGAGCAT |
| *mcr-1*-pUC20_F | TGCGGCAATCATACTGCATCTAGAGTCGACCTGCAG |
| *mcr-1*-pUC20_R | GCCTACTTGCTGAGATGAGAATTCACTGGCCGTCGT |
| pUC20-*mcr-1*_F | TGCAGGTCGACTCTAGATGCAGTATGATTGCCGCAA |
| pUC20-*mcr-1*_R | ACGACGGCCAGTGAATTCTCATCTCAGCAAGTAGGC |
| *dinB*-pBAD44_F | TTTCGCATGCGTTTTTGAGCGCCGATGGTAGTGTGG |
| *dinB*-pBAD44_R | CGCATCGGTATGCCCGGTCACCGGCGCCACAGGTGC |
| pBAD44-*dinB*_F | GCACCTGTGGCGCCGGTGACCGGGCATACCGATGCG |
| pBAD44-*dinB*_R | CCACACTACCATCGGCGCTCAAAAACGCATGCGAAA |
| Lib01 | GGCTTTTCAAGACTGAAGTCTAG |
| Lib02 | AAAACAGCATAGCTCTAAAACG |
| Lib03 | AAAACAGCATAGCTCTAAAACA |
| Lib04 | AAAACAGCATAGCTCTAAAACT |
| W1205 | CGGAAGATAAAGTGATGCAGGT |
| W1206 | CCTTCCTGCAACTCGAATTA |
| *modF*_F | GACGAGTGATGTGGGAAAACGTTTTAGAGCTAGAAATAGC |
| *modF*_R | GTTTTCCCACATCACTCGTCACTAGTATTATACCTAGGAC |
| *cysS*_F | TTCAGCCGGAGCCTGATCATGTTTTAGAGCTAGAAATAGC |
| *cysS*_R | ATGATCAGGCTCCGGCTGAAACTAGTATTATACCTAGGAC |
| *hisR*_F | ACAACTGGAATCACAATCCAGTTTTAGAGCTAGAAATAGC |
| *hisR*_R | TGGATTGTGATTCCAGTTGTACTAGTATTATACCTAGGAC |
| *serS*_F | CTGGCCAATGGATTTCGATCGTTTTAGAGCTAGAAATAGC |
| *serS*_R | GATCGAAATCCATTGGCCAGACTAGTATTATACCTAGGAC |
| *serV*_F | TGACCGCATACTCCCTTAGCGTTTTAGAGCTAGAAATAGC |
| *serV*_R | GCTAAGGGAGTATGCGGTCAACTAGTATTATACCTAGGAC |
| *fabI*_F | GTAGGCGATGGATAGTTTGCGTTTTAGAGCTAGAAATAGC |
| *fabI*_R | GCAAACTATCCATCGCCTACACTAGTATTATACCTAGGAC |
| *fabZ*_F | GTTCTTACTGGTGGATCGCGGTTTTAGAGCTAGAAATAGC |
| *fabZ*_R | CGCGATCCACCAGTAAGAACACTAGTATTATACCTAGGAC |
| *accA*_F | TTCGCTTCCAGCTCTGCAATGTTTTAGAGCTAGAAATAGC |
| *accA*_R | ATTGCAGAGCTGGAAGCGAAACTAGTATTATACCTAGGAC |
| *accB*_F | AACTTGCGGCAGGAGCTGCAGTTTTAGAGCTAGAAATAGC |
| *accB*_R | TGCAGCTCCTGCCGCAAGTTACTAGTATTATACCTAGGAC |
| *murG*_F | TTTTGTTCATGAAGTACAACGTTTTAGAGCTAGAAATAGC |
| *murG*_R | GTTGTACTTCATGAACAAAAACTAGTATTATACCTAGGAC |
| *fabA_*F | AAGAAGGTCTTCTTTTGTATGTTTTAGAGCTAGAAATAGC |
| *fabA*_R | ATACAAAAGAAGACCTTCTTACTAGTATTATACCTAGGAC |
| *fabH*_F | CCCAGATTTCACGTATTGATGTTTTAGAGCTAGAAATAGC |
| *fabH*_R | ATCAATACGTGAAATCTGGGACTAGTATTATACCTAGGAC |
| *nuoC*_F | AACAGCATGACGTAAGGTTTGTTTTAGAGCTAGAAATAGC |
| *nuoC*_R | AAACCTTACGTCATGCTGTTACTAGTATTATACCTAGGAC |
| *atpB*_F | AGGCTTGCAGCGTAATGATCGTTTTAGAGCTAGAAATAGC |
| *atpB*_R | GATCATTACGCTGCAAGCCTACTAGTATTATACCTAGGAC |
| *atpF*_F | TCATGCAGAACAGAACGAACGTTTTAGAGCTAGAAATAGC |
| *atpF*_R | GTTCGTTCTGTTCTGCATGAACTAGTATTATACCTAGGAC |
| *purN*_F | TTTAATTTTGTTGGTTTTACGTTTTAGAGCTAGAAATAGC |
| *purN*_R | GTAAAACCAACAAAATTAAAACTAGTATTATACCTAGGAC |
| *dxr*_F | CGTGCTGCAACCAATCGAGCGTTTTAGAGCTAGAAATAGC |
| *dxr*_R | GCTCGATTGGTTGCAGCACGACTAGTATTATACCTAGGAC |
| *ispD*_F | TTGCATTCGACGGCCAAATCGTTTTAGAGCTAGAAATAGC |
| *ispD*_R | GATTTGGCCGTCGAATGCAAACTAGTATTATACCTAGGAC |
| *dinB*_F | AAATTTACGCGCGGGATAATGTTTTAGAGCTAGAAATAGC |
| *dinB*_R | ATTATCCCGCGCGTAAATTTACTAGTATTATACCTAGGAC |
| *fecA*_F | AAAAGCGGAAAACGAGAGACGTTTTAGAGCTAGAAATAGC |
| *fecA*_R | GTCTCTCGTTTTCCGCTTTTACTAGTATTATACCTAGGAC |
| *galR*_F | ATTATTAATGACGCGGGAAAGTTTTAGAGCTAGAAATAGC |
| *galR*_R | TTTCCCGCGTCATTAATAATACTAGTATTATACCTAGGAC |
| *ghxP*_F | CCAGGCGTCGAGTGAACCGCGTTTTAGAGCTAGAAATAGC |
| *ghxP*_R | GCGGTTCACTCGACGCCTGGACTAGTATTATACCTAGGAC |
| *hdeD*_F | AAATATCGCCAGAGACGAACGTTTTAGAGCTAGAAATAGC |
| *hdeD*_R | GTTCGTCTCTGGCGATATTTACTAGTATTATACCTAGGAC |
| *nudK*_F | GTGCAGGGTGAAATAGTTATGTTTTAGAGCTAGAAATAGC |
| *nudK*_R | ATAACTATTTCACCCTGCACACTAGTATTATACCTAGGAC |
| *rraB*_F | CCAGATCGTCTGCGGAAAGAGTTTTAGAGCTAGAAATAGC |
| *rraB*_R | TCTTTCCGCAGACGATCTGGACTAGTATTATACCTAGGAC |
| *xanP*_F | CATCGCCAGCAGATGCTGACGTTTTAGAGCTAGAAATAGC |
| *xanP*_R | GTCAGCATCTGCTGGCGATGACTAGTATTATACCTAGGAC |
| *yafN*_F | GATCAATAAAGTATTTAGCTGTTTTAGAGCTAGAAATAGC |
| *yafN*_R | AGCTAAATACTTTATTGATCACTAGTATTATACCTAGGAC |
| *yafO*_F | GCATCGCGACCAAATATATCGTTTTAGAGCTAGAAATAGC |
| *yafO*_R | GATATATTTGGTCGCGATGCACTAGTATTATACCTAGGAC |
| *ybfc*_F | ATTATTTAAGACAGGAAACGGTTTTAGAGCTAGAAATAGC |
| *ybfc*_R | CGTTTCCTGTCTTAAATAATACTAGTATTATACCTAGGAC |
| *yobH*_F | ACATTGCAAACCTAATCCTGGTTTTAGAGCTAGAAATAGC |
| *yobH*_R | CAGGATTAGGTTTGCAATGTACTAGTATTATACCTAGGAC |

**Supplementary Table S5. Gene information used in this study.**

| Gene | Sequences |
| --- | --- |
| *dcas9_hyper* | ATGGATAAGAAATACTCAATAGGCTTAGCTATCGGCACAAATAGCGTCGGATGGGCGGTGATCACTGATGAATATAAGGTTCCGTCTAAAAAGTTCAAGGTTCTGGGAAATACAGACCGCCACAGTATCAAAAAAAATCTTATAGGGGCTCTTTTATTTGACAGTGGAGAGACAGCGGAAGCGACTCGTCTCAAACGGACAGCTCGTAGAAGGTATACACGTCGGAAGAATCGTATTTGTTATCTACAGGAGATTTTTTCAAATGAGATGGCGAAAGTAGATGATAGTTTCTTTCATCGACTTGAAGAGTCTTTTTTGGTGGAAGAAGACAAGAAGCATGAACGTCATCCTATTTTTGGAAATATAGTAGATGAAGTTGCTTATCATGAGAAATATCCAACTATCTATCATCTGCGAAAAAAATTGGTAGATTCTACTGATAAAGCGGATTTGCGCTTAATCTATTTGGCCTTAGCGCATATGATTAAGTTTCGTGGTCATTTTTTGATTGAGGGAGATTTAAATCCTGATAATAGTGATGTGGACAAACTATTTATCCAGTTGGTACAAACCTACAATCAATTATTTGAAGAAAACCCTATTAACGCAAGTGGAGTAGATGCTAAAGCGATTCTTTCTGCACGATTGAGTAAATCAAGACGATTAGAAAATCTCATTGCTCAGCTCCCCGGTGAGAAGAAAAATGGCTTATTTGGGAATCTCATTGCTTTGTCATTGGGTTTGACCCCTAATTTTAAATCAAATTTTGATTTGGCAGAAGATGCTAAATTACAGCTTTCAAAAGATACTTACGATGATGATTTAGATAATTTATTGGCGCAAATTGGAGATCAATATGCTGATTTGTTTTTGGCAGCTAAGAATTTATCAGATGCTATTTTACTTTCAGATATCCTAAGAGTAAATACTGAAATAACTAAGGCTCCCCTATCAGCTTCAATGATTAAACGCTACGATGAACATCATCAAGACTTGACTCTTTTAAAAGCTTTAGTTCGACAACAACTTCCAGAAAAGTATAAAGAAATCTTTTTTGATCAATCAAAAAACGGATATGCAGGTTATATTGATGGGGGAGCTAGCCAAGAAGAATTTTATAAATTTATCAAACCAATTTTAGAAAAAATGGATGGTACTGAGGAATTATTGGTGAAACTAAATCGTGAAGATTTGCTGCGCAAGCAACGGACCTTTGACAACGGCTCTATTCCCCATCAAATTCACTTGGGTGAGCTGCATGCTATTTTGAGAAGACAAGAAGACTTTTATCCATTTTTAAAAGACAATCGTGAGAAGATTGAAAAAATCTTGACTTTTCGAATTCCTTATTATGTTGGTCCATTGGCGCGTGGCAATAGTCGTTTTGCATGGATGACTCGGAAGTCTGAAGAAACATTTACCCCATGGAATTTTGAAGAAGTTGTCGATAAAGGTGCTTCAGCTCAATCATTTATTGAACGCATGACAAACTTTGATAAAAATCTTCCAAATGAAAAAGTACTACCAAAACATAGTTTGCTTTATGAGTATTTTACGGTTTATAACGAATTGACAAAGGTCAAATATGTTACTGAAGGAATGCGAAAACCAGCATTTCTTTCAGGTGAACAGAAGAAAGCCATTGTTGATTTACTCTTCAAAACAAATCGAAAAGTAACCGTTAAGCAATTAAAAGAAGATTATTTCAAAAAAATAGAATGTTTTGATAGTGTTGAAATTTCAGGAGTTGAAGATAGATTTAATGCTTCATTAGGTACCTACCATGATTTGCTAAAAATTATTAAAGATAAAGATTTTTTGGATAATGAAGAAAATGAAGATATCTTAGAGGATATTGTTTTAACATTGACCTTATTTGAAGATAGGGAGATGATTGAGGAAAGACTTAAAACATATGCTCACCTCTTTGATGATAAGGTGATGAAACAGCTTAAACGTCGCCGTTATACTGGTTGGGGACGTTTGTCTCGAAAATTGATTAATGGTATTAGGGATAAGCAATCTGGCAAAACAATATTAGATTTTTTGAAATCAGATGGTTTTGCCAATCGCAATTTTATGCAGCTGATCCATGATGATAGTTTGACATTTAAAGAAGACATTCAAAAAGCACAAGTGTCTGGACAAGGCGATAGTTTACATGAACATATTGCAAATTTAGCTGGTAGCCCTGCTATTAAAAAAGGTATTTTACAGACTGTAAAAGTTGTTGATGAATTGGTCAAAGTAATGGGGCGGCATAAGCCAGAAAATATCGTTATTGAAATGGCACGTGAAAATCAGACAACTCAAAAGGGCCAGAAAAATTCGCGAGAGCGTATGAAACGAATCGAAGAAGGTATCAAAGAATTAGGAAGTCAGATTCTTAAAGAGCATCCTGTTGAAAATACTCAATTGCAAAATGAAAAGCTCTATCTCTATTATCTCCAAAATGGAAGAGACATGTATGTGGACCAAGAATTAGATATTAATCGTTTAAGTGATTATGATGTCGATGCCATTGTTCCACAAAGTTTCCTTAAAGACGATTCAATAGACAATAAGGTCTTAACGCGTTCTGATAAAAATCGTGGTAAATCGGATAACGTTCCAAGTGAAGAAGTAGTCAAAAAGATGAAAAACTATTGGAGACAACTTCTAAACGCCAAGTTAATCACTCAACGTAAGTTTGATAATTTAACGAAAGCTGAACGTGGAGGTTTGAGTGAACTTGATAAAGCTGGTTTTATCAAACGCCAATTGGTTGAAACTCGCCAAATCACTAAGCATGTGGCACAAATTTTGGATAGTCGCATGAATACTAAATACGATGAAAATGATAAACTTATTCGAGAGGTTAAAGTGATTACCTTAAAATCTAAATTAGTTTCTGACTTCCGAAAAGATTTCCAATTCTATAAAGTACGTGAGATTAACAATTACCATCATGCCCATGATGCGTATCTAAATGCCGTCGTTGGAACTGCTTTGATTAAGAAATATCCAAAACTTGAATCGGAGTTTGTCTATGGTGATTATAAAGTTTATGATGTTCGTAAAATGATTGCTAAGTCTGAGCAAGAAATAGGCAAAGCAACCGCAAAATATTTCTTTTACTCTAATATCATGAACTTCTTCAAAACAGAAATTACACTTGCAAATGGAGAGATTCGCAAACGCCCTCTAATCGAAACTAATGGGGAAACTGGAGAAATTGTCTGGGATAAAGGGCGAGATTTTGCCACAGTGCGCAAAGTATTGTCCATGCCCCAAGTCAATATTGTCAAGAAAACAGAAGTACAGACAGGCGGATTCTCCAAGGAGTCAATTTTACCAAAAAGAAATTCGGACAAGCTTATTGCTCGTAAAAAAGACTGGGATCCAAAAAAATATGGTGGTTTTGATAGTCCAACGGTAGCTTATTCAGTCCTAGTGGTTGCTAAGGTGGAAAAAGGGAAATCGAAGAAGTTAAAATCCGTTAAAGAGTTACTAGGGATCACAATTATGGAAAGAAGTTCCTTTGAAAAAAATCCGATTGACTTTTTAGAAGCTAAAGGATATAAGGAAGTTAAAAAAGACTTAATCATTAAACTACCTAAATATAGTCTTTTTGAGTTAGAAAACGGTCGTAAACGGATGCTGGCTAGTGCCGGAGAATTACAAAAAGGAAATGAGCTGGCTCTGCCAAGCAAATATGTGAATTTTTTATATTTAGCTAGTCATTATGAAAAGTTGAAGGGTAGTCCAGAAGATAACGAACAAAAACAATTGTTTGTGGAGCAGCATAAGCATTATTTAGATGAGATTATTGAGCAAATCAGTGAATTTTCTAAGCGTGTTATTTTAGCAGATGCCAATTTAGATAAAGTTCTTAGTGCATATAACAAACATAGAGACAAACCAATACGTGAACAAGCAGAAAATATTATTCATTTATTTACGTTGACGAATCTTGGAGCTCCCGCTGCTTTTAAATATTTTGATACAACAATTGATCGTAAACGATATACGTCTACAAAAGAAGTTTTAGATGCCACTCTTATCCATCAATCCATCACTGGTCTTTATGAAACACGCATTGATTTGAGTCAGCTAGGAGGTGACTAA |
| *cas1* | ATGGCTGGTTGGCGTACTGTTGTGGTAAATACCCACTCGAAATTATCCTATAAGAATAATCATCTGATTTTTAAGGATGCCTATAAAACGGAGCTGATCCATTTATCAGAAATTGATATTTTGTTATTAGAAACGACCGATATTGTCTTGTCCACTATGCTGGTAAAACGGCTAGTGGATGAGAATGTCCTTGTCATATTCTGTGATGATAAACGATTACCAACAGCTATGCTGATGCCTTTTTATGGTCGTCATGATTCGAGTTTACAGCTTGGGAAACAAATGTCCTGGTCAGAAACAGTCAAATCGCAGGTTTGGACGACGATTATTGCTCAAAAGATTTTGAATCAATCTTGCTATCTAGGAGCATGCTCCTATTTTGAAAAATCCCAATCTATTATGGATTTATATCATGGTTTGGAAAATTTTGATCCGAGTAATCGAGAAGGGCATGCAGCGAGAATTTATTTTAATACACTTTTTGGGAACGATTTCTCAAGAGATTTGGAGCATCCAATCAATGCAGGTCTGGATTATGGTTATACTTTATTATTGAGTATGTTTGCGCGTGAAGTGGTTGTGTCTGGATGTATGACTCAGTTTGGGCTTAAACACGCTAATCAGTTTAATCAGTTCAATTTTGCTAGCGATATTATGGAACCATTTAGGCCTTTAGTGGATAAGATTGTTTATGAAAATCGAAATCAGCCTTTTCCCAAAATAAAGAGAGAGTTATTTACTTTGTTTTCAGATACATTTTCATATAATGGTAAAGAGATGTATCTCACGAATATTATTAGCGATTATACTAAAAAAGTTGTCAAAGCTCTGAATAATGAAGGGAAAGGAGTTCCTGAATTTAGGATATGA |
| *cas2* | ATGAGTTATAGATATATGAGAATGATACTTATGTTTGATATGCCGACGGACACCGCTGAGGAACGAAAAGCCTATCGAAAATTTCGGAAATTTTTACTTAGTGAAGGGTTTATCATGCATCAATTTTCTATTTATAGTAAGTTGCTGTTGAATAATACAGCTAACAATGCCATGATTGGTCGGCTGAGGGAGCATAATCCTAATAAAGGAAATATTACATTACTAACGGTCACGGAAAAACAGTTTGCACGAATGATTTATTTACATGGTGAAAGAAATAATTGTATTGCAAACTCCGATGAAAGACTTGTATTTCTTGGGGAGGCTTTTGATGAATCTTAA |
| *csn2* | ATGAATCTTAATTTTTCCTTACTAGATGAACCGATTCCATTAAGAGGCGGTACAATTCTTGTGCTCGAAGATGTCTGTGTATTTTCAAAAATAGTGCAATATTGTTACCAATATGAGGAAGATTCTGAACTTAAATTTTTTGATCACAAGATGAAAACAATCAAAGAATCAGAAATCATGCTTGTAACAGATATTTTAGGATTTGATGTTAACTCCTCAACCATTTTAAAATTGATTCATGCAGATTTAGAATCTCAATTTAATGAGAAACCCGAAGTGAAATCGATGATTGACAAATTGGTTGCTACGATTACAGAACTGATTGTCTTTGAATGCTTAGAAAATGAATTAGATTTAGAGTATGATGAAATCACAATCCTGGAATTGATTAAGTCCTTAGGAGTAAAAGTAGAAACGCAAAGTGATACTATTTTTGAAAAATGTCTAGAGATACTTCAAATTTTCAAATATCTCACTAAGAAAAAGTTGCTTATTTTTGTCAATAGCGGAGCTTTTCTAACAAAGGATGAAGTGGCTAGTTTACAAGAGTATATATCATTGACAAATTTAACAGTTCTCTTTTTAGAACCACGTGAACTATATGATTTTCCGCAGTATATTTTAGATGAAGATTATTTCTTAATAACTAAAAATATGGTATAA |
| *tracr* | GTTGGAACCATTCAAAACAGCATAGCAAGTTAAAATAAGGCTAGTCCGTTATCAACTTGAAAAAGTGGCACCGAGTCGGTGCTTTTTTT |
| CRISPR repeat | GTTTTAGAGCTATGCTGTTTTGAATGGTCCCAAAAC |
| J23119 promoter | TTGACAGCTAGCTCAGTCCTAGGTATAATACTAGT |
| gRNA scaffold | GTTTTAGAGCTAGAAATAGCAAGTTAAAATAAGGCTAGTCCGTTATCAACTTGAAAAAGTGGCACCGAGTCGGTGC |
| homologous arm  of *dinB* | CAGCGCGAAGATTATGCCGTTCTTTAAAACGCTGCTGGTGGAGCTCGCGCCAGTGTTCGACTCGCTCGATAATAAAATTATTATTACCGGGCATACCGATGCGATGGCCTACAAAAACAATATCTACAACAACTGGAACCTTTCGGGTGACCGCGCGCTTTCGGCTCGTCGGGTGCTGGAAGAGGCCGGAATGCCGGAAGATAAAGTGATGCAGGTAAGCGCAATGGCGGACCAGATGCTGCTGGATTCCAAAAATCCGCAAAGCGCGGGCAACCGGCGCATTGAGATTATGGTGCTGACCAAAAGTGCGTCCGATACGCTGTATCAATACTTTGGTCAGCATGGGGATAAAGTGGTGCAGCCGCTGGTGCAAAAGCTGGATAAGCAGCAGGTGCTTTCGCAGCGAACGCGTTAAATGCTGAATCTTTACGCATTTCTCAAACCCTGAAATCACTGTATACTTTACCAGTGTTGAGAGGTGAGCAATGCGTAAAATCATTTGAATTTATATTATGAATAACATACAAATAAGAAACTATCAGCCTGGCGATTTTCAGCAACTATGCGCTATTTTCATTAGAGCGGTTACGATGACCGCCAGTCAGCATTATTCACCACAACAAATTTCCGCCTGGGCGCAGATTGACGAATCTCGCTGGAAGGAGAAACTCGCGAAATCACAAGTGTGGGTTGCGATCATTAATGCACAACCGGTTGGTTTTATTTCCCGCATTGAACATTATATCGATATGTTATTTGTTGACCCTGAATACACCCGCCGTGGGGTTGCCAGCGCTTTGTTAAAACCTTTGATTAAGTCTGAATCCGAACTTACGGTGGACGCAAGCATAACCGCAAAACCCTTTTTTGAACGTTATGGTTTTCAGACAGTTAAGCAGCAGCGCGTTGAATGCCGGGGAGCGTGGTTTACTAATTTTTATATGCGATATAAACCGCAACATTAAATCCAGCTTGCAATGAAAATAACGCCCGCCTGGTA |
| *dinB* operon | ATGCGTAAAATCATTCATGTGGATATGGACTGCTTTTTCGCCGCAGTGGAGATGCGCGACAATCCCGCCCTGCGCGATATCCCTATTGCTATTGGCGGCAGCCGCGAACGTCGGGGGGTGATCAGCACCGCCAATTATCCCGCGCGTAAATTTGGCGTACGTAGCGCTATGCCGACAGGGATGGCGCTCAAATTATGCCCACATCTCACCTTGCTTCCGGGGCGCTTTGACGCCTACAAAGAAGCCTCAAATCATATCCGTGAAATCTTCTCGCGCTACACCTCGCGCATTGAACCGTTGTCACTGGATGAGGCTTATCTCGATGTCACCGATAGCGTCCATTGCCACGGTTCTGCGACCCTCATCGCCCAGGAAATCCGCCAGACAATCTTCAACGAGCTGCAACTGACGGCGTCTGCGGGCGTGGCACCAGTAAAGTTTCTCGCCAAAATCGCCTCCGACATGAATAAACCCAACGGCCAGTTTGTGATTACGCCGGCAGAAGTTCCGGCATTTTTACAAACCTTACCGCTGGCAAAAATCCCCGGCGTCGGCAAAGTCTCAGCGGCAAAACTGGAAGCGATGGGGCTGCGGACCTGCGGTGATGTACAAAAGTGTGATCTGGTGATGCTGCTTAAACGCTTTGGCAAATTTGGCCGCATTTTGTGGGAGCGTAGTCAGGGGATTGACGAACGCGATGTTAACAGCGAACGGTTGCGAAAATCCGTCGGCGTGGAACGCACGATGGCGGAAGATATTCATCACTGGTCTGAATGTGAAGCGATTATCGAGCGGCTGTATCCGGAACTTGAACGCCGTCTGGCAAAGGTAAAACCTGATTTACTGATTGCTCGCCAGGGGGTGAAATTAAAGTTCGACGATTTTCAGCAAACCACCCAGGAGCACGTCTGGCCGCGGCTGAATAAAGCTGATCTAATCGCCACCGCGCGTAAAACCTGGGATGAACGCCGCGGCGGGCGCGGTGTGCGTCTGGTGGGGCTGCATGTGACGTTGCTTGACCCGCAAATGGAAAGACAACTGGTGCTGGGATTATGATGTATACTATTATGTATATTCTGGTGTGCATTATTATGAGGGTATCACTGTATGCATCGAATTCTCGCTGAAAAATCGGTCAATATCACTGAGTTACGTAAAAACCCAGCTAAATACTTTATTGATCAACCGGTTGCGGTTCTTTCTAATAATCGCCCCGCAGGATATCTCTTAAGTGCCAGCGCATTCGAAGCGTTAATGGACATGCTTGCTGAACAAGAGGAGAAAAAGCCCATAAAGGCGCGCTTCCGTCCAAGTGCTGCAAGATTAGAGGAAATTACACGCCGCGCTGAACAATATCTTAATGATATGACGGATGATGATTTCAATGACTTTAAGGAATAAGGATGCGGGTATTCAAAACAAAACTTATTCGCCTGCAACTTACAGCAGAGGAACTTGATGCGTTAACGGCGGATTTTATTTCCTATAAGCGTGACGGTGTTTTGCCAGATATATTTGGTCGCGATGCACTCTACGACGACTCCTTTACCTGGCCATTAATCAAATTTGAGCGAGTTGCTCATATTCATCTGGCAAATGAGAATAATCCATTTCCGCCACAGTTGCGCCAATTCAGCAGAACGAATGACGAAGCGCATTTGGTATATTGTCAGGGGGCGTTTGATGAGCAAGCATGGTTGCTCATTGCCATTCTGAAACCTGAACCTCATAAACTGGCTCGAGATAACAACCAAATGCATAAAATTGGGAAAATGGCAGAAGCGTTTCGCATGCGTTTTTGA |
| *dinB* promoter | TGGAGATTGTTCCCCAGGGATTACGCGTGCTGATTAAAGACGACCAGAACCGCAATATGTTTGAACGCGGCAGCGCGAAGATTATGCCGTTCTTTAAAACGCTGCTGGTGGAGCTCGCGCCAGTGTTCGACTCGCTCGATAATAAAATTATTATTACCGGGCATACCGATGCGATGGCCTACAAAAACAATATCTACAACAACTGGAACCTTTCGGGTGACCGCGCGCTTTCGGCTCGTCGGGTGCTGGAAGAGGCCGGAATGCCGGAAGATAAAGTGATGCAGGTAAGCGCAATGGCGGACCAGATGCTGCTGGATTCCAAAAATCCGCAAAGCGCGGGCAACCGGCGCATTGAGATTATGGTGCTGACCAAAAGTGCGTCCGATACGCTGTATCAATACTTTGGTCAGCATGGGGATAAAGTGGTGCAGCCGCTGGTGCAAAAGCTGGATAAGCAGCAGGTGCTTTCGCAGCGAACGCGTTAAATGCTGAATCTTTACGCATTTCTCAAACCCTGAAATCACTGTATACTTTACCAGTGTTGAGAGGTGAGCAATGCGTAAAATCATTCATGTGGATATGGACTGCTTTTTCGCCGCA |
| *ccdB* | ATGTCTCAATTTACGCTATATAAAAACAAAGATAAAAGTTCAGCTAAAACCTACCCATATTTTGTTGATGTTCAAAGTGATCTACTTGATAACTTAAATACTCGATTAGTCATTCCATTAACACCAATCGAACTACTTGATAAGAAAGCACCAAGTCACCTTTGTCCTACGATTCATATTGATGAAGGTGACTTTATAATGCTGACTCAACAAATGACAAGTGTTCCAGTTAAAATCTTATCCGAACCTGTTAATGAGTTATCGACCTTCAGAAATGAGATTATTGCTGCAATTGATTTTTTAATCACTGGCATTTAA |
| *dnaE* | ATGATCAAAATAGCCACACGTAAATATTTAGGCAAACAAAATGTCTATGACATTGGAGTTGAGCGCGACCATAATTTTGCACTCAAAAATGGCTTCATAGCTTCTAATTGTTTCAATTGTTTAAGCTATGAAACGGAAATATTGACAGTAGAATATGGATTATTACCGATTGGTAAAATTGTAGAAAAGCGCATCGAATGTACTGTTTATAGCGTTGATAATAATGGAAATATTTATACACAACCTGTAGCACAATGGCACGATCGCGGAGAACAAGAGGTGTTTGAGTATTGTTTGGAAGATGGTTCATTGATTCGGGCAACAAAAGACCATAAGTTTATGACTGTTGATGGTCAAATGTTGCCAATTGATGAAATATTTGAACGTGAATTGGATTTGATGCGGGTTGATAATTTGCCGAATTGA |
| *tra Ori* | GACCCAGGCGCTCGGTCTTGCCTTGCTCGTCGGTGATGTACTTCACCAGCTCCGCGAAGTCGCTCTTCTTGATGGAGCGCATGGGGACGTGCTTGGCAATCACGCGCACCCCCCGGCCGTTTTAGCGGCTAAAAAAGTCATGGCTCTGCCCTCGGGCGGACCACGCCCATCATGACCTTGCCAAGCTCGTCCTGCTTCTCTTCGATCTTCGCCAGCAGGGCGAGGATCGTGGCATCACCGAACCGCGCCGTGCGCGGGTCGTCGGTGAGCCAGAGTTTCAGCAGGCCGCCCAGGCGGCCCAGGTCGCCATTGATGCGGGCCAGCTCGCGGACGTGCTCATAGTCCACGACGCCCGTGATTTTGTAGCCCTGGCCGACGGCCAGCAGGTAGGCCGACAGGCTCATGCCGGCCGCCGCCGCCTTTTCCTCAATCGCTCTTCGTTCGTCTGGAAGGCAGTACACCTTGATAGGTGGGCTGCCCTTCCTGGTTGGCTTGGTTTCATCAGCCATCCGCTTGCCCTCATCTGTTACGCCGGCGGTAGCCGGCCAGCCTCGCAGAGCAGGATTCCCGTTGAGCACCGCCAGGTGCGAATAAGGGACAGTGAAGAAGGAACACCCGCTCGCGGGTGGGCCTACTTCACCTATCCTGCCCGGCTGACGCCGTTGGATACACCAAGGAAAGTCTACACGAACCCTTTGGCAAAATCCTGTATATCGTGCGAAAAAGGATGGATATACCGAAAAAATCGCTATAATGACCCCGAAGCAGGGTTATGCAGCGGAAA |

**References**

[1] D. Zhang, S. Xu, Y. Wang, P. Bin, G. Zhu, *Int. J. Mol. Sci.* **2021**, *22*, 9245.

[2] H. Zhang, B. Chen, Z. Wang, K. Peng, Y. Liu, Z. Wang, *Microbiol. Spectr.* **2024**, *12*, e03884.

[3] X. Lu, X. Xiao, Y. Liu, Y. Li, R. Li, Z. Wang, *Int. J. Antimicrob. Agents* **2019**, *54*, 99.

[4] H. Zhang, Y. Li, Y. Jiang, X. Lu, R. Li, D. Peng, Z. Wang, Y. Liu, *Microbiol. Spectr.* **2021**, *9*, e0064821.
